# Embryo model completes gastrulation to neurulation and organogenesis

**DOI:** 10.1038/s41586-022-05246-3

**Published:** 2022-08-25

**Authors:** Gianluca Amadei, Charlotte E. Handford, Chengxiang Qiu, Joachim De Jonghe, Hannah Greenfeld, Martin Tran, Beth K. Martin, Dong-Yuan Chen, Alejandro Aguilera-Castrejon, Jacob H. Hanna, Michael B. Elowitz, Florian Hollfelder, Jay Shendure, David M. Glover, Magdalena Zernicka-Goetz

**Affiliations:** 1 Department of Physiology, Development and Neuroscience, University of Cambridge, Cambridge, UK; 2 Division of Biology and Biological Engineering, California Institute of Technology, Pasadena, CA USA; 3 Centre for Trophoblast Research, University of Cambridge, Cambridge, UK; 4 Department of Genome Sciences, University of Washington, Seattle, WA USA; 5 Department of Biochemistry, University of Cambridge, Cambridge, UK; 6 Department of Molecular Genetics, Weizmann Institute of Science, Rehovot, Israel; 7 Allen Discovery Center for Cell Lineage Tracing, Seattle, WA USA; 8 Brotman Baty Institute for Precision Medicine, Seattle, WA USA; 9 Howard Hughes Medical Institute, Seattle, WA USA; 10 Present Address: Department of Biology, University of Padua, Padua, Italy; 11 Present Address: Francis Crick Institute, London, UK

**Keywords:** Gastrulation, Neurulation, Embryonic stem cells, Organogenesis, Stem-cell differentiation

## Abstract

Embryonic stem (ES) cells can undergo many aspects of mammalian embryogenesis in vitro^1–5^, but their developmental potential is substantially extended by interactions with extraembryonic stem cells, including trophoblast stem (TS) cells, extraembryonic endoderm stem (XEN) cells and inducible XEN (iXEN) cells^6–11^. Here we assembled stem cell-derived embryos in vitro from mouse ES cells, TS cells and iXEN cells and showed that they recapitulate the development of whole natural mouse embryo in utero up to day 8.5 post-fertilization. Our embryo model displays headfolds with defined forebrain and midbrain regions and develops a beating heart-like structure, a trunk comprising a neural tube and somites, a tail bud containing neuromesodermal progenitors, a gut tube, and primordial germ cells. This complete embryo model develops within an extraembryonic yolk sac that initiates blood island development. Notably, we demonstrate that the neurulating embryo model assembled from *Pax6*-knockout ES cells aggregated with wild-type TS cells and iXEN cells recapitulates the ventral domain expansion of the neural tube that occurs in natural, ubiquitous *Pax6*-knockout embryos. Thus, these complete embryoids are a powerful in vitro model for dissecting the roles of diverse cell lineages and genes in development. Our results demonstrate the self-organization ability of ES cells and two types of extraembryonic stem cells to reconstitute mammalian development through and beyond gastrulation to neurulation and early organogenesis.

## Abstract (web summary)

Synthetic mouse embryos assembled from embryonic stem cells, trophoblast stem cells and induced extraembryonic endoderm stem cells closely recapitulate the development of wild-type and mutant natural mouse embryos up to embryonic day 8.5.

## Main

In natural development, the zygote develops into the epiblast, which will form the organism; the extraembryonic visceral endoderm (VE), which contributes to the yolk sac; and the extraembryonic ectoderm (ExE), which contributes to the placenta. Stem cells corresponding to these three lineages offer the possibility to completely regenerate the mammalian organism from multiple components, instead of from a single totipotent zygote.

ES cells, which are derived from the epiblast, show a remarkable ability to form embryo-like structures upon aggregation and, when embedded in Matrigel, can be induced to form trunk-like structures with somites, a neural tube and a gut^1–5^. Although neural development can be promoted in such ‘gastruloids’ by inhibiting the initial burst of Wnt activity, they do not accurately replicate gastrulation movements, nor do they represent the complete anatomy of natural embryos. Other model embryoids generated from ES cells aggregated with an ectopic morphogen signalling centre can develop the posterior midbrain, neural tube, cardiac tissue and gut tube only^3^. Thus, these models do not recapitulate the entirety of development to neurulation.

Signals originating from extraembryonic tissues are essential to pattern the epiblast and drive the establishment of the anterior–posterior axis. Guided by this requirement, we have assembled embryoids by aggregating ES cells with TS cells derived from ExE precursors and XEN cells derived from VE precursors^7,11^. Substituting XEN cells with ES cells that transiently express the VE master regulator GATA4 (iXEN cells) improved the efficiency and developmental potential of the resulting ETiX embryoids^10^. ETiX embryoids specify an anterior organizer, the anterior visceral endoderm (AVE), which migrates to position the primitive streak to initiate gastrulation movements^10^ that are essential for subsequent development.

Here we combine our previous method—in which ETiX embryoids complete the morphological events of gastrulation^10^—with methods for post-implantation mouse embryo culture^12,13^ to show that ETiX embryoids can develop beyond neurulation to the equivalent of natural embryos 8.5 days post-fertilization. Specifically, they establish all brain regions, a neural tube, a beating heart and a gut tube. The neural tube is flanked by developing somites and primordial germ cells (PGCs) form in the tail region. This complete embryo model develops within an extraembryonic yolk sac that forms blood islands. Thus, gastrulating and neurulating embryoids offer a powerful, physiologically relevant model of post-implantation embryogenesis.

## Development through neurulation

**Fig. 1:**
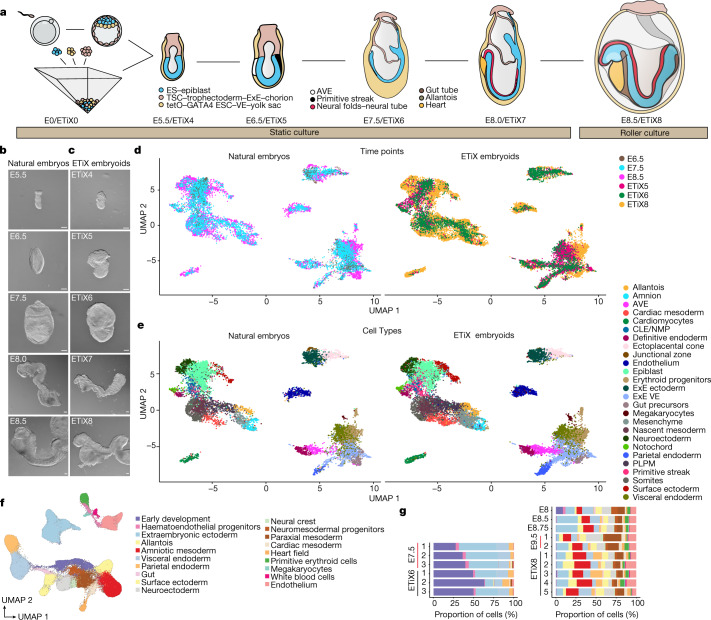
ETiX embryoids recapitulate developmental milestones of the natural mouse embryo up to E8.5. **a**, Schematic of ETiX embryoid formation. ETiX embryoids are formed by aggregating ES cells, TS cells and ES cells transiently expressing GATA4, and by day 4 they generate structures that resemble post-implantation stage natural E5.5 embryos. They subsequently develop to gastrulation (E6.5/ETiX day 5 (ETiX5)) and neurulation (E8.0/ETiX day 7 (ETiX7)) stages before initiating organogenesis (E8.5/ETiX day 8 (ETiX8)). **b**,**c**, Bright-field images of natural mouse embryos (**b**) and ETiX embryoids (**c**) at different timepoints highlighting morphological similarities (*n* = 1,197 ETiX4, 237 ETiX5, 170 ETiX6, 100 ETiX7 and 40 ETiX8, from 17 independent experiments). Scale bars, 100 μm. **d**, Uniform manifold approximation and projection (UMAP) analysis of scRNA-seq data at indicated timepoints for natural embryos at E6.5, E7.5 and E8.5 and ETiX embryoids at days 5, 6 and 8 (*n* = 29 ETiX5, 10 ETiX6, 7 ETiX8, 12 E6.5, 14 E7.5 and 9 E8.5) analysed by inDrops sequencing. **e**, Single-cell inDrops RNA-seq UMAP annotated to show cell types identified in natural embryos and ETiX embryoids. AVE, anterior visceral endoderm; CLE, caudal lateral epiblast; NMP, neuromesodermal progenitors; PLPM, posterior lateral plate mesoderm. **f**, Annotated and combined UMAP of natural embryos cultured ex utero and collected at indicated timepoints (E7.5, E8, E8.5, E8.75 and E9.5) and ETiX embryoids (day 6 and day 8) individually labelled and analysed by tiny-sci-RNA-seq. *n* = 8 natural embryos ranging from E7.5 to E9.5, *n* = 3 ETiX6, 2 failed ETiX6, 5 ETiX8 and 4 failed ETiX8. Source data

To examine anterior brain development in ETiX embryoids, we seeded ES cells, TS cells and iXEN cells and allowed them to self-assemble; on day 4, we transferred the ETiX embryoids with correct post-implantation morphology to suspension culture (Fig. 1a). Typically, we set up 2–4 wells of an AggreWell plate to obtain between 100 and 150 embryo-like structures on day 4, in which cavitated epithelial ES cell and TS cell compartments are enveloped by a VE-like layer. These well-organized structures constitute 10–15% of all the structures recovered from any given well. This variability reflects the random collisions between the three types of stem cells in a microwell and the variation in expression of distinct cadherins between these cell types^11^. On day 5, ETiX embryoids that had a proamniotic cavity (resulting from the merger of cavities in the ES cell and TS cell compartments), a fully migrated AVE (at the boundary of the ES cell and TS cell compartments), and were gastrulating (as revealed by the epithelial-to-mesenchymal transition and formation of a cell layer between the ES cells and VE-like layers) were further cultured under conditions that can support the development of embryos ex utero beyond embryonic day (E)7.5^12,13^. This included supplementing the medium with glucose on day 7 and transferring gastrulating embryoids to rotating culture bottles for one additional day (from day 7 to day 8) (Fig. 1a and Methods).

**Extended Data Fig. 1:**
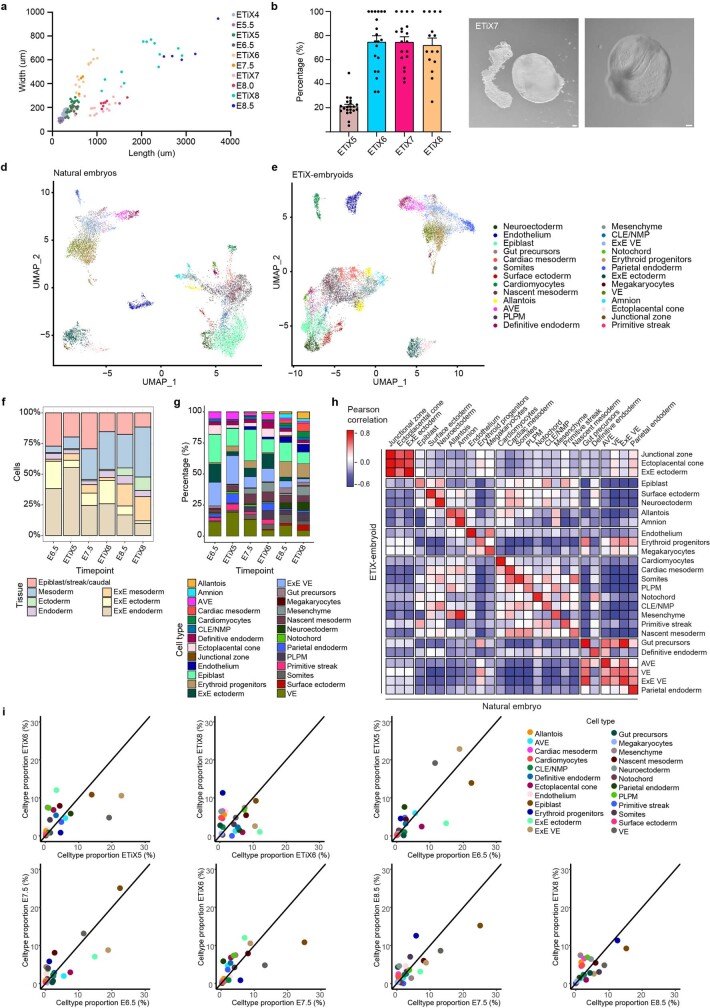
ETiX embryoids develop to comparable sizes and show gene expression patterns similar to natural embryos with reproducible efficiency. **a**. Quantification of ETiX embryoid and natural embryo dimensions at comparable developmental timepoints (n=42 for ETiX4, 24 for ETiX5, 14 for ETiX6, 18 for ETiX7, 12 for ETiX8, 32 for E5.5, 18 for E6.5, 3 for E7.5, 8 for E8.0, 5 for E8.5, from 30 independent experiments). **b**. Quantification of ETiX embryoid formation efficiency from day 5 to day 8 (n = 1197 ETiX4, 237 for ETiX5, 170 for ETiX6, 100 for ETiX7, 40 for ETiX8, from 17 independent experiments). Error bars represent the S.E.M. **c**. Brightfield images of ETiX embryoids recovered after static culture at day 7 prior to dissection to highlight presence of yolk sac. Scale bar, 100 μm (n = 100 for ETiX7, from 17 independent experiments) **d**,**e**. Separated UMAPs of natural embryos (d) and ETiX embryoids (e) analysed by inDrops scRNA-seq. **f**. Stacked column graph binning all sequenced cells in natural embryos and ETiX embryoids according to germ layer and embryonic and extraembryonic origin at indicated timepoints (inDrops scRNA-seq). **g**. Stacked column graph highlighting the proportions of tissue types that emerge during natural embryo and ETiX embryoid development (inDrops scRNA-seq). **h**. Pearson correlation matrices showing global level of similarity across all identified tissues in natural embryos (rows) in comparison to ETiX embryoids (columns) (inDrops scRNA-seq). **i**. Pairwise visualizations of cell-type proportions between natural embryos and ETiX embryoids (inDrops scRNA-seq) Source data

Gastrulating ETiX embryoids strongly resembled natural gastrulating embryos (Fig. 1b,c), although ETiX embryoids displayed greater size variability (Extended Data Fig. 1a). The efficiency of ETiX embryoid development from day 4 to day 5 was on average 21%. Of the structures selected at day 5 for further culture, the efficiency of transitioning from day 5 to day 6, from day 6 to day 7 and from day 7 to day 8 was over 70% at each transition (Extended Data Fig. 1b). Notably, on day 7, neurulating ETiX embryoids cultured in stationary conditions displayed an anterior–posterior axis with bifurcating neural folds extending into a neural tube and culminating in a tail bud, a morphology that resembles the early headfold stage of an E8.0 natural embryo. Posterior to this, the tail bud joined with allantois tissue which connected to the developing chorion (Fig. 1c). The embryoid, allantois and chorion were contained in a fluid-filled sac, which is equivalent to the yolk sac (Extended Data Fig. 1c). Thus, these conditions enabled ETiX embryoids to develop through and beyond gastrulation to neurulation.

To monitor development by examining changes in gene expression at single-cell resolution, we isolated ETiX embryoids at day 5, day 6 and day 8, and natural embryos dissected at E6.5, E7.5 and E8.5 (*n* = 29 for ETiX5, 10 for ETiX6, 7 for ETiX8, 12 for E6.5, 14 for E7.5 and 9 for E8.5), dissociated them into single cells and performed single-cell RNA sequencing (scRNA-seq) using the inDrops method^14–16^ (Methods). UMAP analyses revealed a similar contribution of cells to the developing lineages in natural embryos and ETiX embryoids (Fig. 1d). To determine cell types, the cell populations were subclustered using Seurat and subsequently annotated on the basis of published datasets^17^ (Fig. 1e). We identified 26 cell types on the basis of gene expression patterns, all of which were clearly represented in both natural embryos and ETiX embryoid datasets. Individual clustering of natural embryos versus ETiX embryoids showed similar local cluster topography in the UMAP (Extended Data Fig. 1d,e). Only one cluster in natural embryos was not represented in ETiX embryoids. This missing cluster corresponded to the junctional zone of the placental cluster of the natural embryo—during development, this cell population gives rise to trophoblast giant cells and spongiotrophoblast^18,19^. Some other cell types, notably PGCs and neural crest cells, were not detectable by scRNA-seq in either natural embryos or ETiX embryoids, but were observed by immunofluorescence.

Natural embryos and ETiX embryoids displayed a largely conserved distribution of cells between the different germ layers of the epiblast (ectoderm, mesoderm and endoderm) and between embryonic and extraembryonic lineages (epiblast, ExE, extraembryonic mesoderm and extraembryonic endoderm) (Extended Data Fig. 1f). As expected, natural embryos exhibited an increase in cell-type complexity over time, corresponding to the formation of differentiated tissues and organs. For instance, cardiomyocytes and neuroectoderm emerged starting from E7.5. This increase in cell-type complexity and spatiotemporal maturation of all the identified populations was similar between natural and ETiX embryoids, indicating that the neurulating embryoids followed a similar developmental timeline (Extended Data Fig. 1g). For example, both systems showed the development of the three germ layers and their derivatives (neuroectoderm, surface ectoderm and gut tube progenitors), the beginning of organogenesis (cardiomyocytes), and the formation of extraembryonic tissues such as amnion and allantois. A Pearson correlation matrix indicated a high similarity of gene expression between the cell-type clusters of natural embryos and ETiX embryoids (Extended Data Fig. 1h). Cell-type proportion comparisons at the different timepoints between natural embryos and ETiX embryoids showed gross similarity in individual clusters, although some variability was also observed (Extended Data Fig. 1i). Thus, neurulating embryoids recapitulate the generation of the multiple tissues of the neurulating embryo, as evident from both their morphology and their pattern of cell-type-specific gene expression.

**Extended Data Fig. 2:**
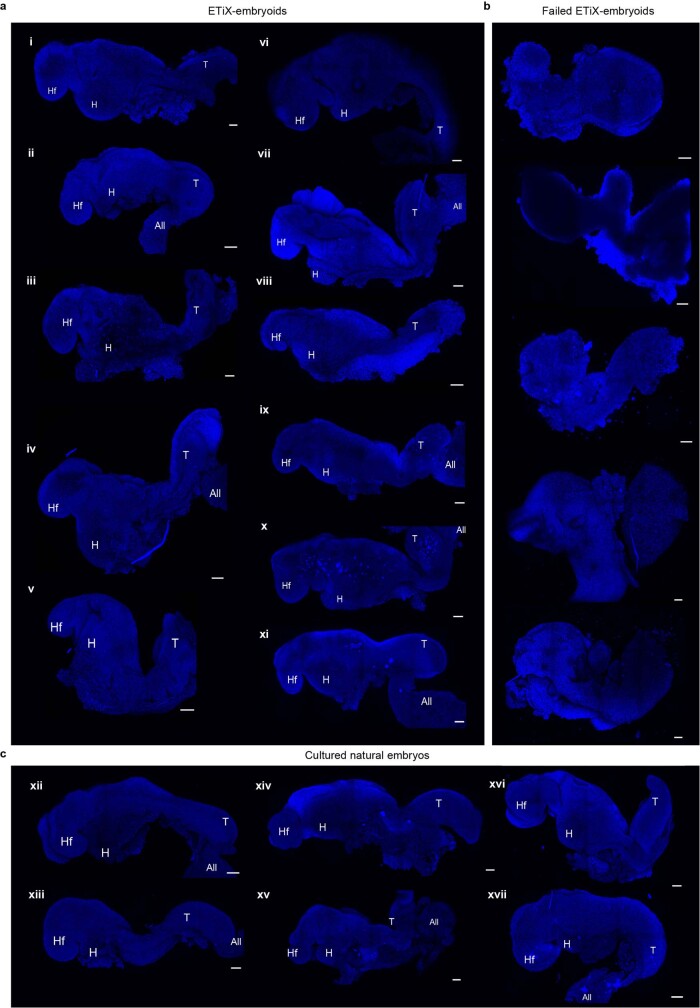
Examples of well-formed ETiX embryoids, failed ETiX embryoids and cultured natural embryos. **a**. Typical day 8 ETiX embryoids judged as developing successfully. **b**. Typical failed ETiX embryoids at day 8. **c**. Natural embryos cultured *ex utero* from E6.5 to E8.5. All structures are stained with DAPI. Morphological features: Hf, Headfolds; H, Heart; T, Tail: All, Allantois. (Some of these samples are also shown in the following panels: v in Fig. 4g; vi in 2d; viii in Ext. Data Fig. 7g; xi in Ext. Data Fig. 4a; xii in Ext. Data Fig. 5c; xiii in Fig. 4b; xvi in Fig. 4h; and xvii in Fig. 2e).

To further assess the reproducibility of neurulating embryoid formation, we performed an additional round of single-cell sequencing in which individual ETiX embryoids of apparently correct morphology, as well as individual natural embryos cultured in vitro from E6.5 and collected at different times in development were individually barcoded and analysed by tiny-sci-RNA-seq^20^ (hereafter referred to as ‘tiny-sci’; Methods), a combinatorial indexing-based method for single-nucleus RNA-sequencing profiling from small amounts of starting material. Furthermore, to understand why some ETiX embryoids did not develop well, we also included individual examples of morphologically aberrant embryoid development at day 6 and day 8 (examples of ‘failed’ embryoids, well-formed ETiX embryoids and natural embryos cultured in vitro (Extended Data Fig. 2)).

**Extended Data Fig. 3:**
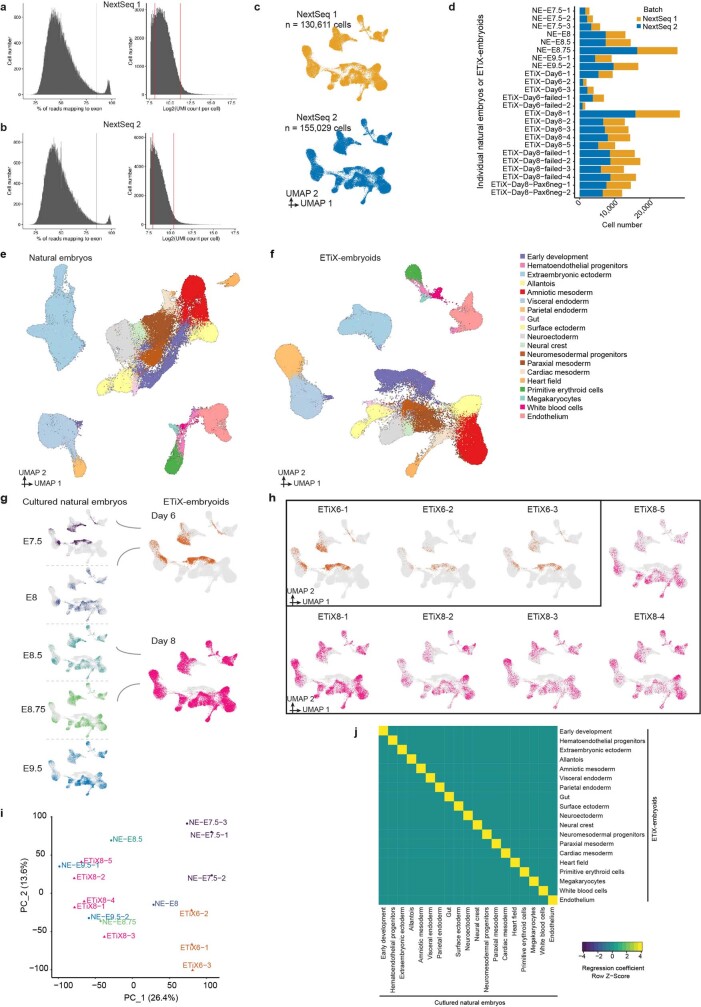
Analysis of ETiX embryoids and natural embryos by tiny sci-RNA-seq. **a**,**b**. Quality control for the first (a) and second replicate (b) of the tiny sci-RNA-seq. Cells with an abnormal percentage of reads mapping to an exon, and too high or too low UMI counts per cell were removed. **c**. UMAP of the first and second replicate of the tiny sci-RNA-seq. The total number of cells in each dataset is indicated. **d**. Batch variation for the first and second replicate of tiny sci-RNA-seq. **e**,**f**. Separated UMAPs of natural embryos (e) and ETiX embryoids (f) analysed with tiny sci-RNA-seq. **g**. Contribution of each timepoint to the global UMAP of the tiny sci-RNA-seq. **h**. Individual UMAPs of standard ETiX embryoids analysed at day 6 and day 8 with tiny sci-RNA-seq. **i**. PCA analysis of all the natural embryo and standard ETiX- mbryoid samples analysed with tiny sci-RNA-seq. **j**. Correlation matrix following a nonnegative least-squares (NNLS) regression analysis showing global level of similarity across all identified tissues in natural embryos (columns) in comparison to ETiX embryoids (rows) in the samples analysed by tiny sci-RNA-seq Source data

**Extended Data Fig. 4:**
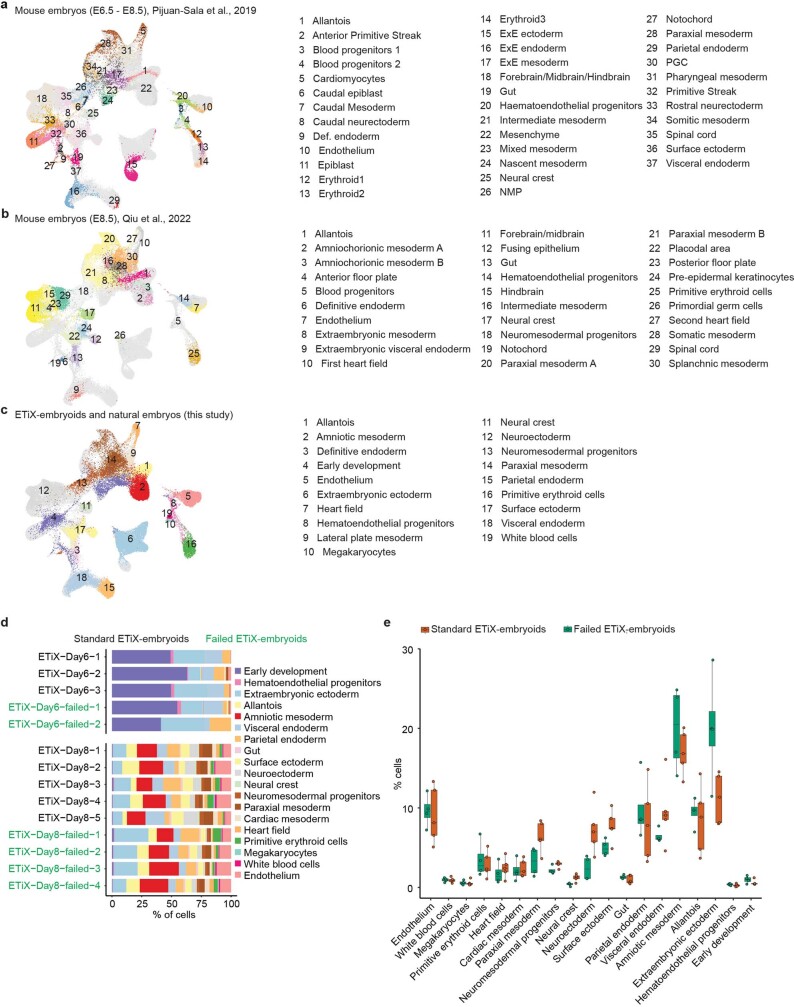
Tiny sci-RNA-seq dataset integrates seamlessly with published single cell sequencing datasets and highlights differences between well-formed and failed ETiX embryoids. **a**,**b**,**c**. Integration of the tiny sci-RNA-seq dataset generated in this study with two published single cell sequencing mouse datasets^17,21^. **d**. Cell type proportions for each individual well-formed (standard) ETiX embryoid and each “failed” ETiX embryoid (classified through aberrant morphology) sequenced at D6 and D8 with tiny sci-RNA-seq (n = 3 for standard ETiX6, 2 for failed ETiX6, 5 for standard ETiX8, 4 for failed ETiX8 from 2 independent experiments). **e**. Averaged cell type proportions for well-formed (standard) and “failed” ETiX embryoids sequenced at D8 with tiny sci-RNA-seq. Cell type proportions from each individual sample are also plotted. In each boxplot the center line shows the medians; the box limits indicate the 25th and 75th percentiles; the whiskers extend to the 5th and 95th percentiles; the replicates are represented by the dots. (n = 5 for standard ETiX8, 4 for failed ETiX8 from 2 independent experiments) Source data

After data processing and quality control, this new dataset contained profiles for 285,640 cells and showed no discernible batch effect between sequencing rounds (Extended Data Fig. 3a–d). Annotation of this dataset yielded 19 clusters that were present both in natural embryos and ETiX embryoids (Fig. 1f). Notably, in contrast to our previous dataset, we could clearly detect the presence of a population of neural crest cells but we were still unable to detect PGCs. In this dataset, individual clustering of natural embryos versus ETiX embryoids also showed similar local cluster topography in the UMAP (Extended Data Fig. 3e,f). Individual ETiX embryoids had very similar cell-type composition from sample to sample (Fig. 1g). From the UMAPs at different timepoints, it was apparent that day 6 and day 8 ETiX embryoids were most similar to E7.5 and E8.5 or E8.75 natural embryos, respectively (Extended Data Fig. 3g), a finding that we also observed with individual replicates (Extended Data Fig. 3h). The similarity between natural embryos and ETiX embryoids was also confirmed by principal component analysis, which showed the samples to be arranged by developmental age along PC1, whereby day 6 ETiX embryoids most closely resembled E7.5 natural embryos and day 8 ETiX embryoids most closely resembled E8.5–E8.75 natural embryos (Extended Data Fig. 3i). Notably, we found E9.5 natural embryos intermingled with these samples, suggesting that the natural embryos did not substantially develop ex utero beyond E8.75. Day 6 ETiX embryoids and E7.5 natural embryos separated along PC2, but day 8 ETiX embryoids and E8.5–9.5 natural embryos did not (Extended Data Fig. 3i). To assess the overall similarity of each specific cluster to every other cluster in the dataset, we analysed the dataset with a non-negative least-squares (NNLS) regression matrix (Extended Data Fig. 3j) (Methods). This revealed that every cluster in the natural embryo dataset showed highest similarity with its ETiX embryoid counterpart (for example, the natural heart field cluster was most similar to the ETiX embryoid heart field cluster; Extended Data Fig. 3j). Furthermore, this tiny-sci dataset integrated very well with published datasets^17,21^, confirming that we had captured the same populations as previously reported in our sampling of natural embryos and ETiX embryoids (Extended Data Fig. 4a–c). Differences between good ETiX embryoids and failed structures were not readily apparent, since the overall cell composition of even the failed embryoids with aberrant morphology appeared very similar to well-formed embryoids and natural embryos (Extended Data Fig. 4d), perhaps reflecting some limitations of RNA-sequencing analyses and/or a failure of morphological events despite the continuation of appropriate gene expression. However, we note that the failed structures tended to have a smaller proportion of paraxial mesoderm, neuroectoderm and surface ectoderm at the expense of a larger proportion of ExE cells (Extended Data Fig. 4e).

Next, to identify global transcriptional differences between natural embryos and ETiX embryoids, we performed Gene Ontology analysis. Natural embryos showed an enrichment of terms associated with uterine development, implantation and remodulation of the endothelial compartment, whereas ETiX embryoids showed terms associated with embryonic morphogenesis (Supplementary Tables 1–4). Gene Ontology analysis did not indicate an obvious stress signature or metabolic differences between natural embryos and ETiX embryoids.

## Development of fore- and midbrain regions

In natural development, the anterior side of the epiblast retains its epithelial character, up-regulates the neuroectodermal marker *Sox1* from E8.0, and begins the formation of the nervous system^22^. The neuroectodermal lineage gives rise to the forebrain, midbrain, hindbrain and the spinal cord.

**Fig. 2:**
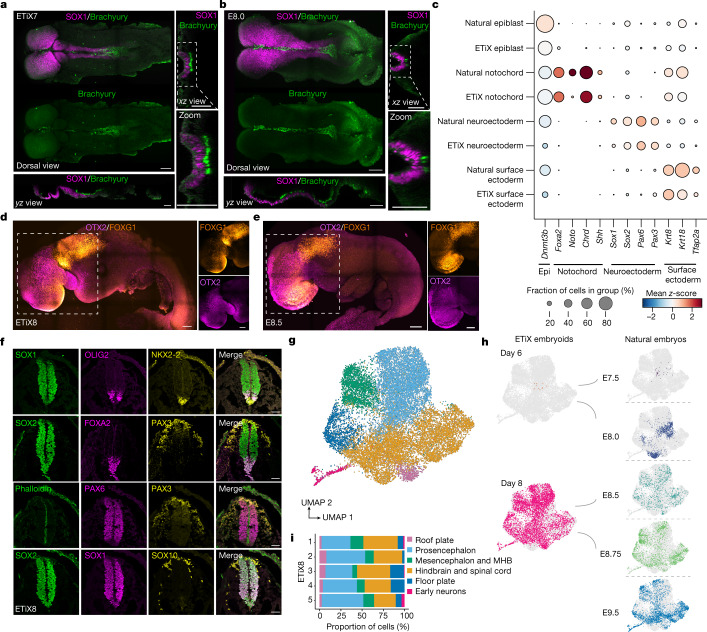
ETiX embryoids develop anterior brain and patterned neural tube. **a**,**b**, Ventral view (main image) of day 7 ETiX embryoids after static culture (**a**) and E8.0 natural embryo (**b**) showing SOX1-positive neural folds and neural tube extending from the anterior towards the Brachyury-positive posterior. Optical *yz* (bottom) and *xz* (right) sections show notochord lying below the neural tube (*n* = 11 day 7 ETiX embryoids from 4 experiments, *n* = 3 E8.0 embryos). Scale bars, 100 μm **c**, Dot plot showing average levels and proportion of cells expressing indicated genes in selected tissues of natural embryos and ETiX embryoids (from inDrops scRNA-seq data). Epi, epiblast. **d**,**e**, Lateral view of day 8 ETiX embryoid (**d**) and E8.5 natural embryo (**e**) showing FOXG1 in telencephalon and OTX2 in forebrain and midbrain (*n* = 4 ETiX8 from 3 experiments; *n* = 2 E8.5 embryos). Scale bars, 100 μm. **f**, Coronal view of neural tube sections showing dorso–ventral patterning in day 8 ETiX embryoids. Sections reveal pan-neural markers (SOX1 and SOX2), dorsal markers (PAX6 and PAX3), ventral markers (FOXA2, OLIG2 and NKX2-2) and neural crest markers (SOX10 and PAX3). Scale bars, 50 μm. *n* = 3 ETiX8 from 3 experiments. **g**, Subclustered UMAP of neural progenitors highlighting neural subtypes from tiny-sci-RNA-seq. **h**, Individual UMAPs showing the contribution of each timepoint to global UMAP in **g**. **i**, The proportion of cell types in **g** in each individual day 8 ETiX embryoid sequenced by tiny-sci-RNA-seq. MHB, midbrain–hindbrain boundary. Source data

**Extended Data Fig. 5:**
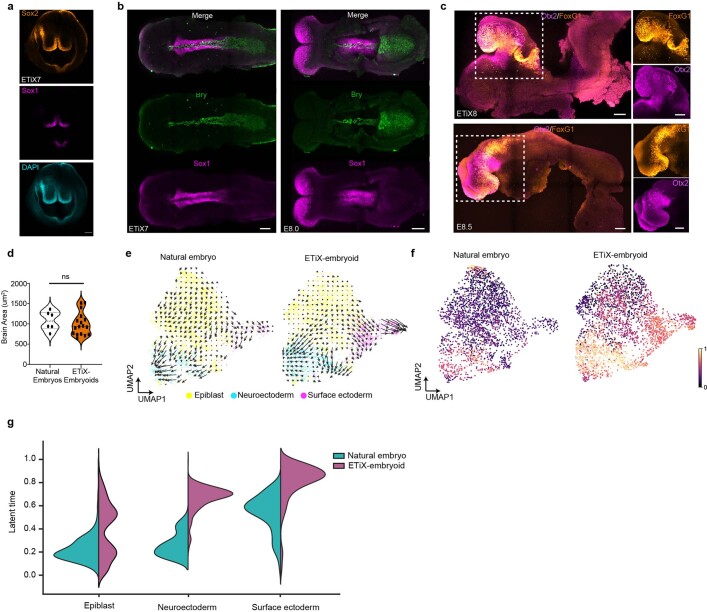
ETiX embryoids show neural folds and a developing tail bud with comparable differentiation trajectory and timing. **a**. Day 7 ETiX embryoid recovered after stationary culture stained to reveal SOX2, SOX1 and DNA highlighting formation of the rostral neural folds (n = 11 ETiX7 from 4 independent experiments, n = 3 E8.0 natural embryos). **b**. Dorsal views of day 7 ETiX embryoid recovered after stationary culture (left) and natural E8.0 embryo (right) showing formation of SOX1 positive neural folds and BRY-positive notochord and tail bud (n = 11 ETiX7 from 4 independent experiments, n = 3 embryos). Scale bar, 100 μm. **c**. Lateral view of day 8 ETiX embryoid (top) and E8.5 natural embryo (bottom) showing FOXG1 expression in the telencephalon and OTX2 restricted to the forebrain and midbrain (n = 4 ETiX8 from 3 independent experiments, n = 2 embryos). Scale bar for a to c,100 μm. **d**. Quantification of brain area in E8.5 natural embryos and day 8 ETiX-embryoids. OTX2 was used to delineate the measured area. Data presented as violin plots with median and quartiles, each dot represents a sample (n = 6 E8.5 embryos and n = 17 ETiX8 from 7 independent experiments). Data are presented as violin plots with median and quartiles. Two-sided unpaired t-test, ns = p > 0.05 (p = 0.5223). **e**. Velocity plots for epiblast, neuroectoderm, surface ectoderm for all time points analysed in the inDrops sequencing dataset. **f**. Latent time analysis for epiblast, neuroectoderm, surface ectoderm for all time points analysed in the inDrops sequencing dataset. **g**. Quantification of the latent time analysis for epiblast, neuroectoderm, surface ectoderm for all time points analysed in the inDrops sequencing dataset Source data

To examine neural development in embryoids, we analysed the expression of well-established neuroectodermal markers by immunofluorescence. SOX1 and SOX2 were expressed in the neuroepithelial cell population, along the entire anteroposterior axis of neurulating embryoids at day 7 in a pattern similar to the natural E8.0 embryo (Fig. 2a,b and Extended Data Fig. 5a,b). The SOX1-positive neural tube tissue made up two thirds by length of the neurulating embryoid at day 7 and culminated in two neural folds (Extended Data Fig. 5a) whereas the SOX1-negative, Brachyury-positive posterior exhibited a tail bud-like morphology (Fig. 2a,b) reminiscent of natural E8.0 embryos. A Brachyury-positive notochord^23^ running below the neural tube was readily apparent in both neurulating natural embryos and ETiX embryoids (Fig. 2a,b).

Next, we examined the scRNA-seq data for the expression of key markers of neuroectoderm; surface ectoderm—which gives rise to most epithelial tissues; notochord—which is important for patterning of the neuroectoderm and neural tube; and epiblast—which is the precursor to all these tissues. In the ETiX embryoids and natural embryos, we observed similar expression of *Foxa2*, *Chordin* and *Shh* marking the notochord^23,24^ (Fig. 2c). Moreover, natural embryos and ETiX embryoids expressed similar levels of *Sox1*, *Sox2*, *Pax6* and *Pax3* in the neuroectoderm, and displayed a similar surface ectoderm signature of keratin gene expression^25^. Thus, tissue-specific gene expression patterns of the neurulating embryoids strongly resemble those of natural embryos.

We found that the transcription factor OTX2, which contributes to patterning of the midbrain and forebrain^26^ showed restricted expression in the anterior-most third of the headfolds of neurulating day 8 ETiX embryoids (Fig. 2d,e). This region corresponds to the forebrain and midbrain of the natural mouse embryo at E8.5^22,27^. The neurulating day 8 embryoids also expressed the transcription factor FOXG1—which has a vital role in brain development—in the same region as in natural E8.5 embryos^28^ (Fig. 2d,e and Extended Data Fig. 5c). The area of the brain demarcated by OTX2 expression was similar in natural embryos and ETiX embryoids (Extended Data Fig. 5d). The neural tube of neurulating day 8 embryoids was closed and showed distinct neural progenitor domains within the neural tube^29–31^, delineated by the expression of the markers PAX6, OLIG2 and NKX2-2^32–34^ (Fig. 2f). FOXA2 was expressed in cells lining the ventral midline of the neural tube, marking a floor plate cell population^35,36^ (Fig. 2f), whereas PAX3 was expressed in the dorsal neural tube, in the somatic mesoderm and in neural crest cells^37^ (Fig. 2f). SOX10 expression confirmed the identity of neural crest cells^38^, which were displaced from the neural tube, as though undergoing the delamination and migration that occurs during natural brain development^38^ (Fig. 2f). We next sought to determine whether formation of neuroectoderm and surface ectoderm occurs in a similar manner in neurulating embryoids in comparison to natural embryos. To this end, we computed transcriptional trajectories using RNA velocity, which integrates the ratios of spliced and unspliced RNAs over time to infer how a starting population evolves and differentiates^39^. Comparison of RNA velocities between natural embryos and neurulating embryoids indicated similar differentiation trajectories from the epiblast to neuroectoderm and surface ectoderm, suggesting that specification of these two tissues follows a similar developmental transcription programme in these two systems (Extended Data Fig. 5e). To determine whether these tissues were forming at comparable times, we performed latent time analysis which assigns an arbitrary combined pseudotime to give a measure of when specific tissues or subpopulations emerge. Latent time analysis indicated that neuroectoderm appeared to be specified later in neurulating embryoids than in natural embryos; this was also observed with surface ectoderm, although this difference was markedly smaller (Extended Data Fig. 5f,g). Together, these findings suggest that neuroectoderm and surface ectoderm are specified with similar transcriptional trajectories in the neurulating ETiX embryoids and natural embryos but with somewhat different timing.

**Extended Data Fig. 6:**
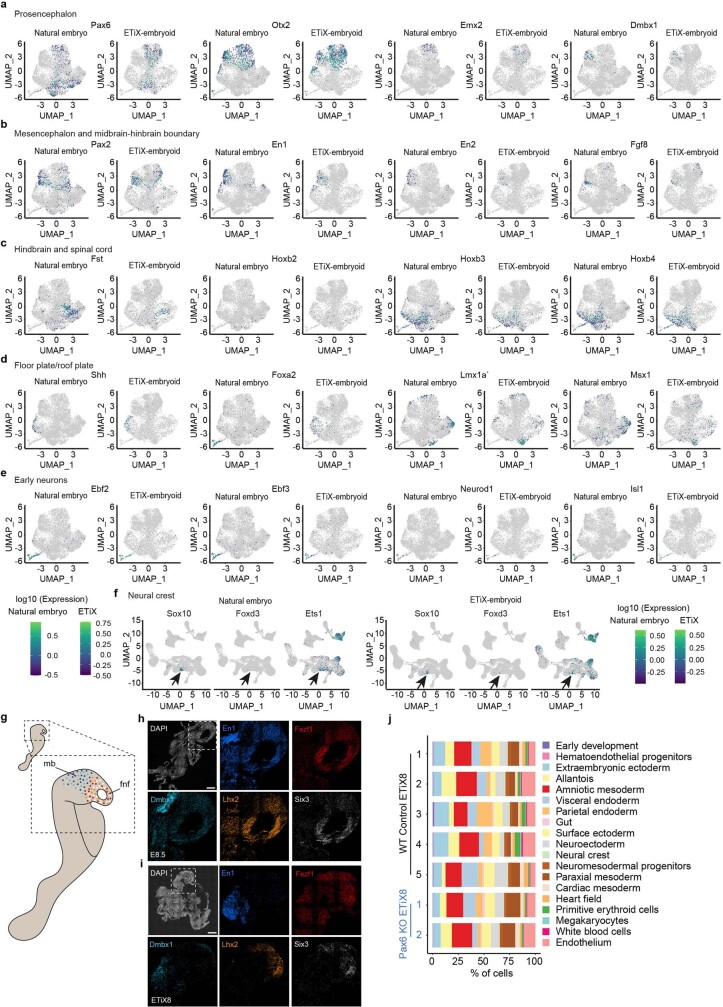
Expression of selected markers for the annotation of neural tissue and localised expression of transcripts revealed by sequential single molecule FISH in natural embryos and ETiX embryoids. Expression of selected gene markers in annotated clusters shown in Fig. 2g. Gene markers of cell populations representing the prosencephalon (**a**), mesencephalon and midbrain-hindbrain boundary (**b**), hindbrain and spinal cord (**c**) floor plate/roof plate (**d**), early neurons (**e**) and neural crest (**f**) are indicated (tiny sci-RNA-seq). **g**. Schematic representation of sample sectioning for smFISH and expected expression pattern of selected genes. **h**,**i**. smFISH panel of n = 1 natural embryo cultured *ex utero* from E6.5 to E8.5 (b) and n = 1 day 8 ETiX-embryoid (c) Scale bar = 200 μm. **j**. The proportion of cell types annotated in Fig. 1f and present in each individual day 8 ETiX embryoid and Pax6 knockout ETiX embryoid sequenced by tiny sci-RNA-seq are shown Source data

We next sought to use the tiny-sci dataset further to explore the different neural cell types present in neurulating ETiX embryoids. Assignment of these subclusters to specific neural identities was performed using well-established markers^21,40^ (Extended Data Fig. 6a–e). Subclustering and annotation of all neuroectoderm-derived cell types showed the presence of cells expressing markers of the hindbrain and spinal cord, prosencephalon, mesencephalon and midbrain–hindbrain boundary. We also observed cells expressing genes indicative of roof plate and floor plate identity (Fig. 2g–i) but without spatial organization data, we cannot conclude that there is dorso–ventral patterning in the brain. We were able to observe the presence of similar neural populations in neurulating ETiX embryoids to those we observed in embryos at E8.5 (Fig. 2g). These neural types were almost completely absent at E7.5, in line with the major burst of neural induction taking place at E8.0. Accordingly, we did not observe neural cells in day 6 ETiX embryoids, which are very similar to E7.5 embryos, whereas in day 8 ETiX embryoids, all these neural subtypes were present, closely matching E8.5 and E8.75 natural embryos (Fig. 2h). Finally, examination of individual ETiX embryoids at day 8 showed that the presence of these neural subtypes was largely replicated in each structure (Fig. 2i). The early neuron population, however, was represented in 3 out of 5 samples examined, suggesting that the specimens with neurons might have been at a slightly more advanced stage with respect to the onset of neurogenesis. Neural crest cells expressed known marker *Ets1*^41^ in addition to *Sox10* (Extended Data Fig. 6f). To confirm that the formation of all these neural subtypes was accompanied by a regionalized pattern of gene expression, we performed sequential single-molecule fluorescence in situ hybridization (smFISH) on a sectioned natural embryo and ETiX embryoid (Extended Data Fig. 6g–i). In both we observed expression of *Fezf1*, *Lhx2* and *Six3* in the forebrain, and also localized expression of *En1* and *Dmbx1* in the midbrain, in agreement with published results^40^.

**Fig. 3:**
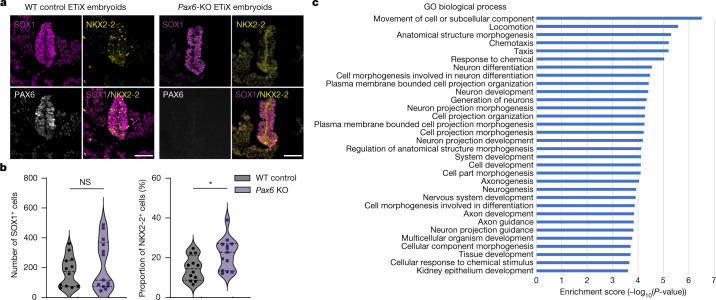
*Pax6* knockout in ETiX embryoids recapitulates known mouse embryonic phenotypes. **a**, Coronal sections of wild-type (WT) and *Pax6*-knockout (KO) ETiX embryoids stained to reveal dorsal and ventral neural tube markers. Scale bar, 50 μm. **b**, Quantification of images represented in **a**, showing no significant difference in SOX1-positive cell number in the neural tube but an increased proportion of NKX2-2-positive cells following *Pax6*-knockout (3 control day 8 ETiX and 4 day 8 *Pax6*-KO ETiX from 3 experiments). Violin plots show median and quartiles. Two-sided Mann–Whitney *U*-test, *P < 0.05. For SOX1-positive cells, *P* = 0.5382; for NKX2-2 positive cells, *P* = 0.0135. **c**, Gene Ontology (GO) analysis of genes enriched in 2 *Pax6-*knockout ETiX embryoids at day 8 (tiny-sci-RNA-seq) compared with 5 ETiX day 8 controls. NS, not significant (*P* > 0.05). Source data

To test whether the neural tube of neurulating embryoids responds to a developmental challenge in the same way as the neural tube of natural embryos, we generated embryoids from a transgenic ES cell line that does not express PAX6. PAX6 is a transcription factor required for neural tube patterning as well as brain and eye development^42^. *Pax6-*knockout ETiX embryoids had similar cell-type proportions as control structures (Extended Data Fig. 6j). In line with development of *Pax6*-knockout natural embryos^32^, the *Pax6-*knockout embryoids showed no alteration in the total number of SOX1-positive cells in the neurectoderm but showed an increase in the proportion of NKX2-2-positive cells, suggesting an expansion of the ventral domain of the neural tube (Fig. 3a,b). To determine other developmental consequences of the *Pax6* deletion, we examined global changes in transcript levels in the absence of *Pax6* and found enrichment of transcripts associated with neuron formation and the development and formation of axons (Fig. 3c), consistent with previous results^43^.

## Somitogenesis and heart development

**Fig. 4:**
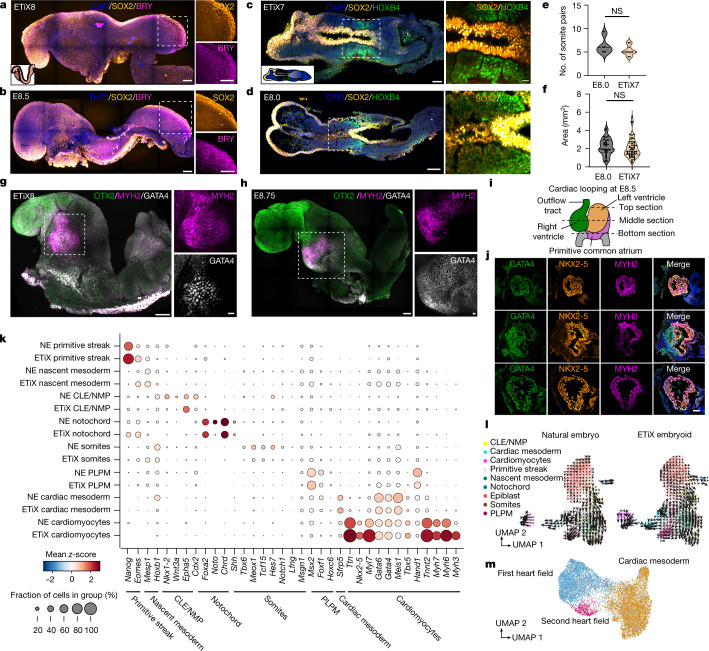
ETiX embryoids undertake somitogenesis and heart formation. **a**,**b**, Lateral view of day 8 ETiX embryoid (**a**) and natural E8.5 embryo (**b**) showing SOX2, Brachyury (BRY) and DNA (DAPI), highlighting NMPs in the tail bud region (*n* = 5 ETiX8 from 4 experiments, *n* = 3 embryos). Scale bars, 100 μm. Inset, schematic view. **c**,**d**, Dorsal view of day 7 ETiX embryoid after stationary culture (**c**) and natural E8.0 embryo (**d**) showing SOX2, HOXB4 and DNA, highlighting somite formation flanking neural tube. Right, magnified view of outlined region containing somites (*n* = 9 day 7 ETiX embryoids from 4 experiments, *n* = 5 E8.0 embryos). Inset, schematic view. Scale bars a-d, 100 μm (main image), 50 μm (magnified view **a**–**c**), 20 μm (magnified view **d**). **e**, Quantification of somite pairs in natural E8.0 embryos and day 7 ETiX embryoids. Violin plots show median and quartiles. Two-sided Mann–Whitney *U*-test, *P* = 0.3020. **f**, Somite area of E8.0 embryos and day 7 ETiX embryoids. Violin plots show median and quartiles. Two-sided Mann–Whitney *U*-test. *P* = 0.2717. For **e**,**f**, *n* = 9 day 7 ETiX embryoids from 4 experiments, *n* = 5 E8.0 embryos. **g**,**h**, Day 8 ETiX embryoid (lateral view) (**g**) and natural E8.75 embryo (**h**) (lateral view) showing OTX2, MYH2 and GATA4, highlighting heart (*n* = 8 ETiX8 from 3 experiments, *n* = 2 natural embryos). Outlined areas are magnified on the right. Scale bars, 100 μm (main image), 20 μm (magnified view). **i**, Schematic of mouse heart at E8.5, indicating location of sections. **j**, Coronal sections of ETiX embryoid at day 8 showing GATA4, NKX2-5 and MYH2. Scale bar, 100 μm. *n* = 3 ETiX8 from 3 independent experiments. **k**, Dot plot showing levels and proportion of cells expressing indicated genes in indicated tissues from natural embryos (NE) and ETiX embryoids by inDrops scRNA-seq. **l**, Velocity plots for epiblast and mesodermal derivatives for time series in the inDrops sequencing dataset. **m**, UMAP of the tiny-sci-RNA-seq dataset, showing cell types in the subclustered cardiac lineage. Source data

**Extended Data Fig. 7:**
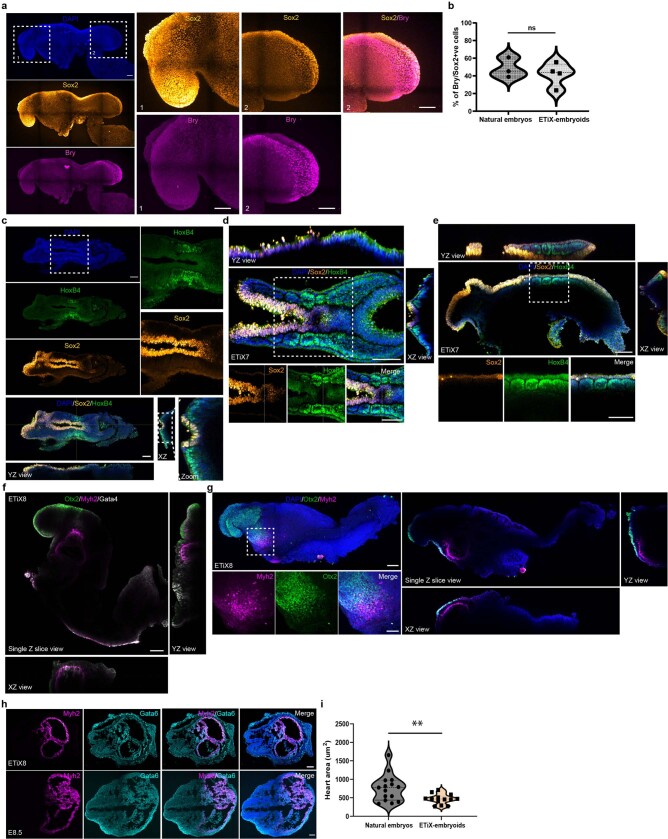
Development of ETiX embryoids mesoderm into somites and cardiac tissue. **a**. Lateral view of day 8 ETiX embryoid shown in Fig. 3a, highlighting the individual channels. Square regions are shown magnified on the right. Scale bars, 100 μm. **b**. Percentages of cells co-expressing BRY and SOX2 in natural embryos and ETiX embryoids. (n = 3 E8.5 embryos and n = 4 ETiX8). Data are presented as violin plots with median and quartiles, each dot represents a sample. Two-sided Mann–Whitney U-test, ns = p > 0.05 (p = 0.5182). **c**. Dorsal view of day 7 ETiX embryoid shown in Fig. 3c, highlighting the individual channels. YZ and XZ views are also shown (n = 9 ETiX7 from 4 experiments, n = 5 E8.0 embryos). **d**. Dorsal and **e**. lateral view of day 7 ETiX embryoid recovered after stationary culture stained to reveal SOX2, HOXB4 and DNA to highlight somite formation flanking the neural tube (n = 9 ETiX7 from 4 independent experiments).YZ and XZ views are also shown. **f**. Orthogonal views of day 8 ETiX-embryoid shown in Fig. 4g. **g**. Lateral view of day 8 ETiX embryoid stained to reveal OTX2, MYH2 and DNA to highlight heart formation (n = 8 ETiX8 from 3 independent experiments). YZ and XZ views are also shown. Scale bar for a-d, 100 μm. Scale bar for magnified region, 50 μm. **h**. Day 8 ETiX embryoid (top) and E8.5 natural embryo (below) sectioned coronally and stained to reveal GATA6 and MYH2 to highlight heart morphogenesis. Scale bar, 200 μm. **i**. Quantifications of the area of the heart in natural embryos and ETiX mbryoids. The MYH2-positive region was utilised to measure the area of the heart or heart-like structure (n = 3 E8.5 embryos, n = 3 ETiX8). Data are presented as violin plots with median and quartiles. Each dot represents a section of the heart and heart-like region. Two-sided unpaired t-test ** = p ≤ 0.01 (exact p value = 0.01) Source data

During natural embryogenesis, NMPs contribute to derivatives of the neural tube and the paraxial mesoderm^44^. To determine whether neurulating ETiX embryoids form NMPs, we performed immunofluorescence to detect the expression of the NMP markers SOX2 and Brachyury in a domain spanning the posterior region of the tail bud of neurulating day 8 ETiX embryoids (Fig. 4a). By contrast, the more anterior regions of day 8 ETiX embryoids expressed SOX2 but not Brachyury—marking the neural lineage—or Brachyury but not SOX2—marking the mesodermal lineage (Fig. 4a and Extended Data Fig. 7a). This pattern of marker expression was similar to that in E8.5 natural embryos (Fig. 4b), consistent with the differentiation trajectory reported for these cells in the embryo^45^, and the co-localization of Brachyury and SOX2 in tail bud cells was similar between ETiX embryoids and natural embryos (Extended Data Fig. 7b).

The paraxial mesoderm in turn gives rise to somites, which are paired blocks of cells that form along the anterior–posterior axis of the embryo and are required for segmental formation of skeletal muscle, blood vessels and skin^46^. Pairs of somites expressing the homeobox protein HOXB4 were observed on either side of the SOX1 and SOX2-positive neural tube population in both day 7 ETiX embryoids and natural E8.0 embryos (Fig. 4c,d and Extended Data Fig. 7c–e). Quantification of the number of somite pairs and the area of somites in ETiX embryoids and natural embryos showed no significant difference between the two (Fig. 4e,f).

A distinct set of cells destined to form the heart also emerges from the primitive streak at gastrulation. In the natural embryo, this developmental event takes place around E8.0 and a heartbeat is established as the cardiac mesoderm differentiates into cardiomyocytes. We observed formation of a beating structure below the encephalon region in day 8 neurulating embryoids (Supplementary Videos 1 and 2). This beating region of the neurulating embryoids expressed myosin heavy chain II (MYH2) and the transcription factor GATA4 (Fig. 4g,h and Extended Data Fig. 7f,g), which are required for cardiac development, in a similar spatiotemporal profile as the natural embryo. Immunostaining of the indicated sections in day 8 neurulating embryoids showed a NKX2-5, GATA4 and MYH2 triple-positive compartment (Fig. 4i,j). The MHY2-positive region also expressed the transcription factor GATA6 (Extended Data Fig. 7h). Comparison with an age-matched E8.5 natural embryo heart showed that the abutting cavities observed in the MYH2-positive region of the ETiX embryoid were very similar to the natural embryo heart, but no clear heart looping was observed. Furthermore, the area of the cardiac domain was decreased in ETiX embryoids compared with natural embryos (Extended Data Fig. 7i).

**Extended Data Fig. 8:**
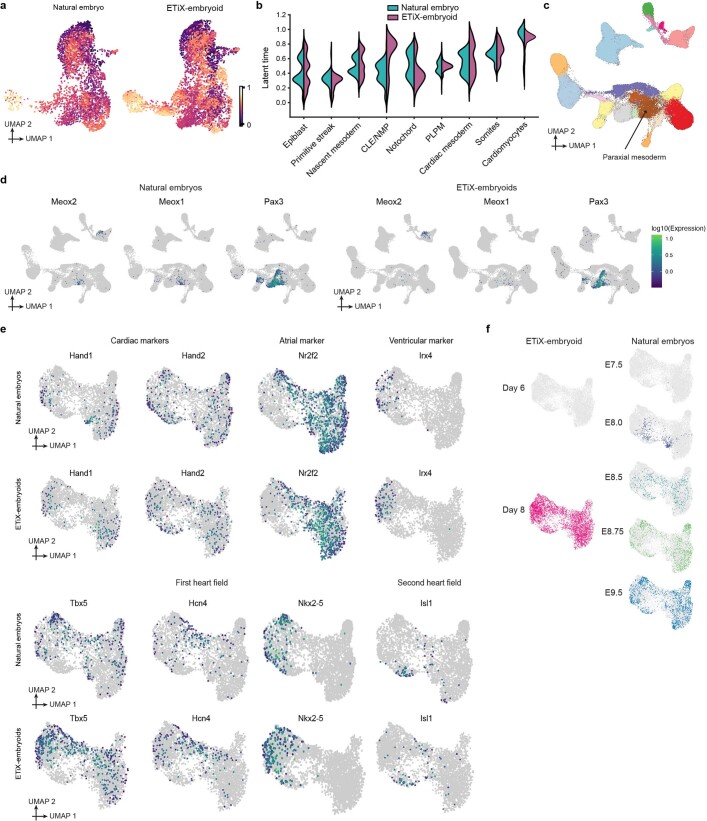
Developmental trajectories, timing of mesoderm differentiation and expression of selected genes. **a**. Latent time for epiblast and mesodermal derivatives for time series in inDrops sequencing dataset. **b**. Quantification of the latent time analysis for epiblast and all the mesodermal derivatives for all time points analysed in the inDrops sequencing dataset. **c**. Annotated UMAP of the tiny sci-RNA-seq dataset highlighting the paraxial mesoderm cluster. **d**. Expression of somite markers *Meox1*, *Meox2* and *Pax3* in natural embryos and ETiX embryoids (tiny sci-RNA-seq dataset). **e**. Expression of cardiac markers *Hand1*, *Hand2*, atrial differentiation marker *Nr2f2*, ventricular differentiation marker *Irx4*, first heart field markers *Tbx5*, *Hcn4*, *Nkx2.5* and second heart field marker *Isl1* in natural embryos and ETiX embryoids (tiny sci-RNA-seq dataset). **f**. UMAP showing individual timepoints for natural embryos and ETiX embryoids in combined UMAP presented in Fig. 4m Source data

The scRNA-seq data from mesoderm and its derivatives corroborated and extended our findings from immunofluorescence (Fig. 4k). The gene expression signature leading to somite formation was apparent in the neurulating embryoids, although the transcript levels of presomitic identity genes^46^ (*Tbx6*, *Hes7* and *Msng1*), Notch pathway genes (*Notch1* and *Lfng*) and a somite marker (*Meox1*) were lower than in natural embryos. Both natural embryos and neurulating embryoids expressed *Gata4* and other important regulators of heart development, including *Gata6*, *Meis1*, *Tbx5* and *Hand1*^17^ (Fig. 4k). Similarly, neurulating embryoids expressed cardiomyocyte markers such as troponin genes (*Ttn* and *Tnnt2*) and myosin genes (*Myh7*, *Myh6*, *Myl3* and *Myl7*), suggesting that mesoderm development in the neurulating embryoids is remarkably similar to that of natural embryos. To further confirm this, we performed RNA velocity analysis on the epiblast and all its mesodermal derivatives on both neurulating embryoids and natural embryos and observed that the differentiation trajectories between the two were very similar (Fig. 4l). Similarly, latent time analysis showed that all these mesodermal derivatives emerge in the neurulating embryoids in a temporal order broadly consistent with that of the natural embryo (Extended Data Fig. 8a). Of the tissues examined, only the caudal lateral epiblast and NMP seemed to emerge at a slightly later time in the neurulating embryoids, whereas notochord formation seemed to occur slightly earlier than in the natural embryo (Extended Data Fig. 8b). We examined the cell cluster derived of paraxial mesoderm in the tiny-sci dataset and found that cultured natural embryos expressed the somite markers *Meox1*, *Meox2* and *Pax3*, whereas ETiX embryoids expressed *Pax3* but instead showed relatively weak *Meox1* or no *Meox2* expression (Extended Data Fig. 8c,d), potentially suggesting differences in somitogenesis between natural embryos and ETiX embryoids.

Using the tiny-sci dataset we analysed the cardiac tissue further to determine whether we could identify additional cell populations. Subclustering of the cardiac lineage (Fig. 4m), which expressed *Hand1* and *Hand2* (Extended Data Fig. 8e), allowed us to identify the first heart field, characterized by robust expression of the canonical markers *Tbx5*, *Nkx2*-*5* and *Hcn4* (Extended Data Fig. 8e), as well as the second heart field, characterized by localized expression of *Isl1* (Extended Data Fig. 8e). We also detected the atrial marker *Nr2f2*^47^ and the ventricular differentiation marker *Irx4*^48^ in both cultured natural embryos and ETiX embryoids (Extended Data Fig. 8e). In agreement with the latent time analysis of the pooled data provided by inDrops sequencing, the tiny-sci dataset also confirmed that cardiac cell types emerged in the neurulating embryoids in a conserved temporal fashion reflecting the developmental sequence of the natural embryo. In fact, we do not observe substantial cardiac lineages in either day 6 ETiX embryoids or the E7.5 natural embryo. Instead, the cardiac lineage of the natural embryo largely emerges from E8.5 onwards and from this perspective, day 8 of ETiX embryoid development captures cell contributions of the natural embryo at E8.5, E8.75 and E9.5 (Extended Data Fig. 8f).

## Initiation of gut development

**Fig. 5:**
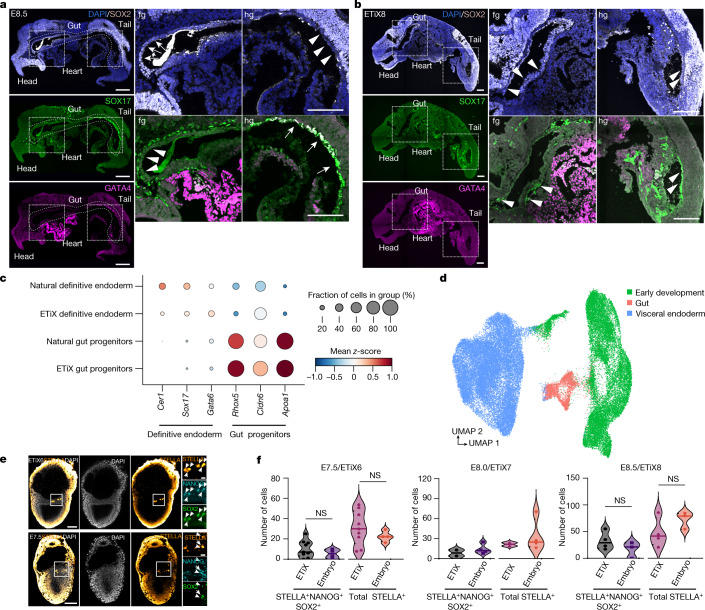
ETiX embryoids develop a gut pocket and primordial germ cells. **a**,**b**, Sagittal sections of natural embryos at E8.5 (**a**) and day 8 ETiX embryoids (**b**) showing SOX2, SOX17 and GATA4. Scale bars, 100 μm. *n* = 3 ETiX8 from 3 experiments, *n* = 2 natural embryos. fg, foregut; hg, hindgut. **c**, Dot plot showing levels and proportion of cells expressing indicated genes in selected tissues of natural embryos and ETiX embryoids by inDrops scRNA-seq. **d**, UMAP of tiny-sci-RNA-seq dataset showing VE, gut and early development cell types. **e**, Day 6 ETiX embryoid (top) and natural E7.5 embryo (bottom) showing STELLA, NANOG and SOX2, highlighting the presence of committed PGCs (*n* = 9 ETiX6 from 2 experiments, *n* = 4 embryos). Outlined regions are magnified on the right. **f**, Quantification of PGCs during ETiX embryoid development (*n* = 9 ETiX6, 4 E7.5, 2 ETiX7, 4 E8.0, 4 ETiX8 and 3 E8.5). PGCs were scored for STELLA, NANOG and SOX2 expression. Violin plots show median and quartiles. Two-sided Mann–Whitney *U*-test, *P* = 0.3375 for ETiX6/E7.5 total STELLA-positive cells, *P* = 0.3042 for ETiX6/E7.5 triple-positive cells, *P* = 0.2277 for ETiX8/E8.5 total STELLA-positive cells, and *P* = 0.2536 for ETiX8/E8.5 triple-positive cells. Source data

**Extended Data Fig. 9:**
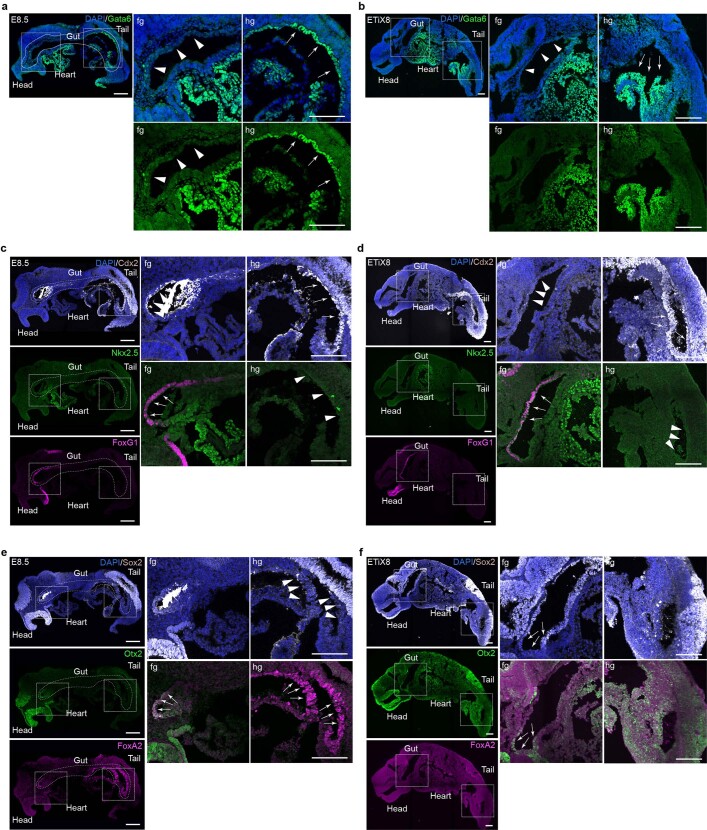
Further characterisation of the gut tube of ETiX embryoids reveals similarities and differences in comparison to natural embryos. **a**,**b**. Sagittal sections of natural embryos at E8.5 (a) and day 8 ETiX embryoids (b) stained to reveal GATA6. (n = 3 ETiX8 from 3 independent experiments, n = 2 embryos). **c**,**d**. Sagittal sections of natural embryos at E8.5 (c) and day 8 ETiX embryoids (d) stained to reveal CDX2, NKX2.5 and FOXG1. (n = 3 ETiX8 from 3 independent experiments, n = 2 embryos). **e**,**f**, Sagittal sections of natural embryos at E8.5 (e) and day 8 ETiX embryoids (f) stained to reveal SOX2, OTX2 and FOXA2. Scale bar for a-f, 100 μm. (n = 3 ETiX8 from 3 experiments, n = 2 embryos).

Having observed extensive development and morphogenesis of ectoderm and mesoderm, we next determined the extent to which neurulating embryoids showed development of definitive endoderm, which gives rise to the gut and associated organs. Sagittal sections of E8.5 natural embryos and day 8 ETiX embryoids revealed the presence of foregut and hindgut pockets (Fig. 5a,b). In addition to being expressed in the brain and neural tube, the transcription factor SOX2 is also expressed in the foregut of natural embryos, a pattern of expression that was conserved in neurulating embryoids (Fig. 5a,b). Similarly, SOX17 was expressed in the hindgut of both natural embryos and neurulating embryoids (Fig. 5a,b) but also in a scattered group of cells suggestive of endothelial precursors^17^. However, the gut tube of the ETiX embryoids did not develop as extensively as natural embryos within the time frame of our observations. Whereas GATA4 was expressed equally prominently in the heart of natural embryos and ETiX embryoids, it was expressed in the hindgut of natural embryos but not of the ETiX embryoids (Fig. 5a,b). By contrast, GATA6 was expressed in the heart and hindgut of both natural embryos and ETiX embryoids (Extended Data Fig. 9a,b). Further characterization showed that the foregut of both ETiX embryoids and natural embryos expressed FOXG1 and OTX2 (Extended Data Fig. 9c–f) and the hindgut of both expressed CDX2 (Extended Data Fig. 9c–f). However, in contrast to the natural embryo, we did not observe any expression of the transcription factor FOXA2 in the gut of ETiX embryoids (Extended Data Fig. 9f). We also did not detect expression of NKX2-5 in natural embryos or in ETiX embryoids, despite its mRNA having been shown to be expressed in the gut^49^ (Extended Data Fig. 9c,d).

**Extended Data Fig. 10:**
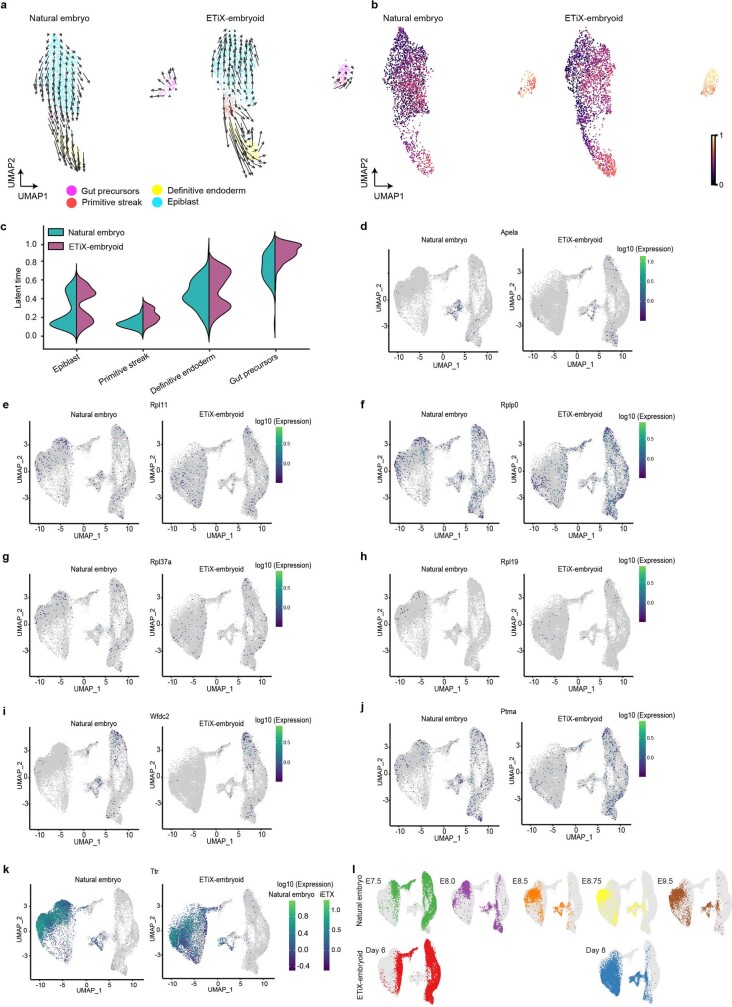
Developmental trajectories and timing of the endoderm and extraembryonic contribution to gut formation. a. Velocity plots of epiblast, definitive endoderm, gut precursors and primitive streak for all time points analysed in the inDrops sequencing dataset. **b**. Latent time analysis of epiblast, definitive endoderm, gut precursors and primitive streak for all time points analysed in the inDrops sequencing dataset. **c**. Quantification of latent time analysis of epiblast, definitive endoderm, gut precursors and primitive streak for all time points analysed in the inDrops sequencing dataset. **d–k**. Expression of selected marker genes of the embryonic (d-j) and extraembryonic (k) endoderm contribution to the gut (tiny sci-RNA-seq dataset). **l**. UMAP showing time series of individual natural embryos and ETiX embryoids to combined UMAP in Fig. 5d Source data

To characterize the gut and associated endodermal tissue further, we turned to our scRNA-seq datasets. The inDrops scRNA-seq data from natural embryos and ETiX embryoids revealed gene expression signatures corresponding to definitive endoderm (*Cer1*^*+*^*Sox17*^*+*^*Gata6*^+^) and VE gut progenitors (*Rhox5*^+^*Cldn6*^*+*^*Apoa1*^*+*^) (Fig. 5c). RNA velocity analysis, however, showed that there are likely to be differences in the differentiation trajectories from epiblast to gut between natural embryos and ETiX embryoids (Extended Data Fig. 10a). By contrast, latent time analysis indicated that the timing of emergence of these lineages was highly similar in embryos and embryoids (Extended Data Fig. 10b,c). Together, these data indicate that gut formation in ETiX embryoids is likely to proceed with similar timing to that in natural embryos, but with some differences in the developmental trajectory. Analysis of the gut and endodermal clusters in the tiny-sci dataset showed that the gut cluster may have both embryonic and extraembryonic contributions (Fig. 5d). We observed higher expression of genes associated with a definitive endoderm origin in the embryonic portion of the gut cluster (Fig. 5d and Extended Data Fig. 10d–j), whereas the presumptive VE-derived portion of the gut cluster (Fig. 5d) expressed higher levels of *Ttr*, which is expressed by gut cells with an extraembryonic origin^49^ (Extended Data Fig. 10k). Cell populations corresponding to precursors of the liver, pancreas, small intestine or colon were not observed in either ETiX embryoids or natural embryos at this stage, suggesting that neither have developed beyond an uncommitted endodermal state under the applied culture conditions, consistent with the onset of organ-specific identities in the gut tube at E8.75^49^. The VE of ETiX embryoids at day 6 was very similar to that of natural embryos at E7.5, but in day 8 ETiX embryoids, cells emerge that appear largely absent in the VE from E8.5 to E9.5 (Extended Data Fig. 10l).

## Development of PGCs

**Extended Data Fig. 11:**
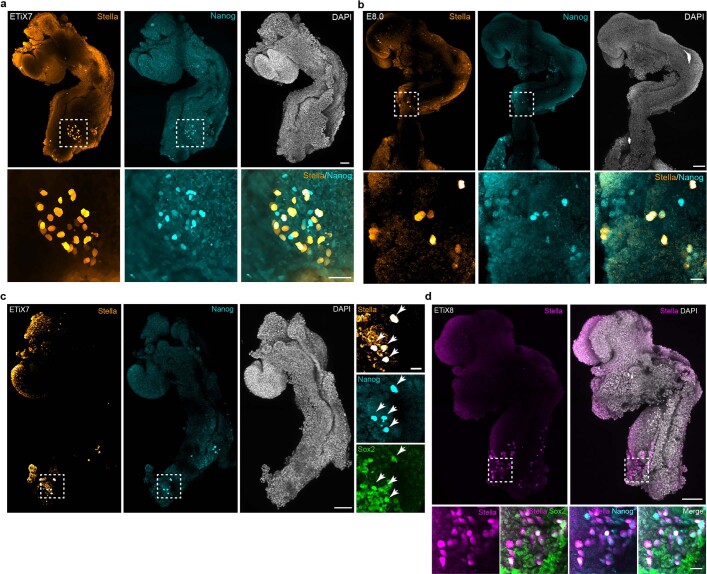
Additional examples of primordial germ cell formation in ETiX embryoids. **a–c** ETiX embryoid (a) at day 7 of development after stationary culture and natural embryo (b) at E8.0 of development stained to reveal STELLA, NANOG and SOX2 to highlight presence of committed PGCs (n = 2 ETiX7 from 2 independent experiments, n = 4 embryos). Boxes are magnified below (a,b) and on the right (c). Scale bars, 100 μm for main panel, 50 μm for magnified boxes. **d**. Day 8 ETiX-embryoid stained to reveal STELLA, NANOG and SOX2 highlighting presence of committed PGCs. Scale bar for a-d, 100 μm. (n = 4 ETiX8 from 3 experiments).

PGCs emerge in the proximo-posterior region of the epiblast, around the same time as Brachyury expression commences in the E6.5 mouse embryo^50,51^. Committed PGCs are characterized by the expression of STELLA, which we detected at the ES cell–TS cell boundary in ETiX embryoids at day 6, similar to the E7.5 natural embryo (Fig. 5e). PGCs were also detectable at later timepoints of ETiX embryoid development (Extended Data Fig. 11a–d). During development, PGCs reactivate the pluripotency markers SOX2 and NANOG as we also observed to occur in ETiX embryoids (Fig. 5e and Extended Data Fig. 11a–d). PGCs were found in proximity to the allantois in ETiX embryoids at days 7 and 8, similar to the natural embryo at E8.0 (Extended Data Fig. 11a–d). Quantification of PGC numbers in natural embryos/ETiX embryoids at E7.5/day 6 and E8.5/day 8 indicated that there were no significant differences between the total number of STELLA^+^ or STELLA^+^NANOG^+^SOX2-positive cells (Fig. 5f). Thus, our inability to detect PGCs in our single-cell datasets probably reflects their very low numbers.

## Yolk sac and blood island development

**Fig. 6:**
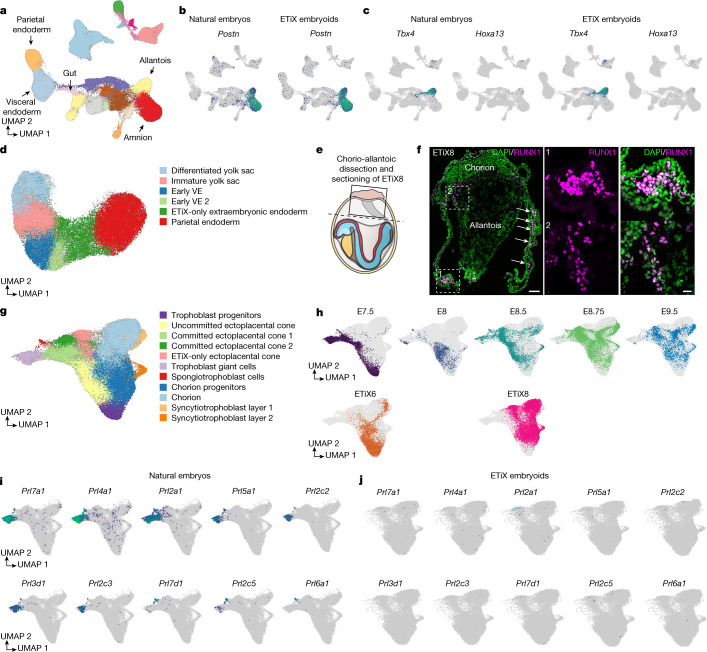
Characterization of extraembryonic lineages in ETiX embryoids. **a**, Global UMAP of the tiny-sci dataset as shown in Fig. 1f; selected cell clusters are highlighted. **b**, Gene expression of the amnion marker periostin (*Postn*) in natural embryos and ETiX embryoids from the tiny-sci-RNA-seq dataset. **c**, Gene expression of the allantois markers *Tbx4* and *Hoxa13* in natural embryos and ETiX embryoids from the tiny-sci-RNA-seq dataset. **d**, Subclustered and annotated UMAP of extraembryonic endoderm from the tiny-sci-RNA-seq dataset. **e**, Schematic of dissection of chorioallantoic attachment of ETiX embryoids. **f**, Left, sagittal section of chorioallantoic attachment and yolk sac of day 8 ETiX embryoid showing RUNX1 and DNA. Arrows highlight blood islands. Outlined regions are magnified in the middle and right panels. *n* = 3 ETiX8 from 3 experiments. Scale bars: 100 μm (left), 20 μm (middle and right). **g**, Subclustered and annotated UMAP of ExE and trophoblast cells from the tiny-sci-RNA-seq dataset. **h**, The contribution of individual timepoints to the subclustered UMAP of the ExE and trophoblast cells. **i**,**j**, Expression of selected prolactin genes in the subclustered UMAP of ExE and trophoblast cells for natural embryos (**i**) and ETiX embryoids (**j**). Source data

**Extended Data Fig. 12:**
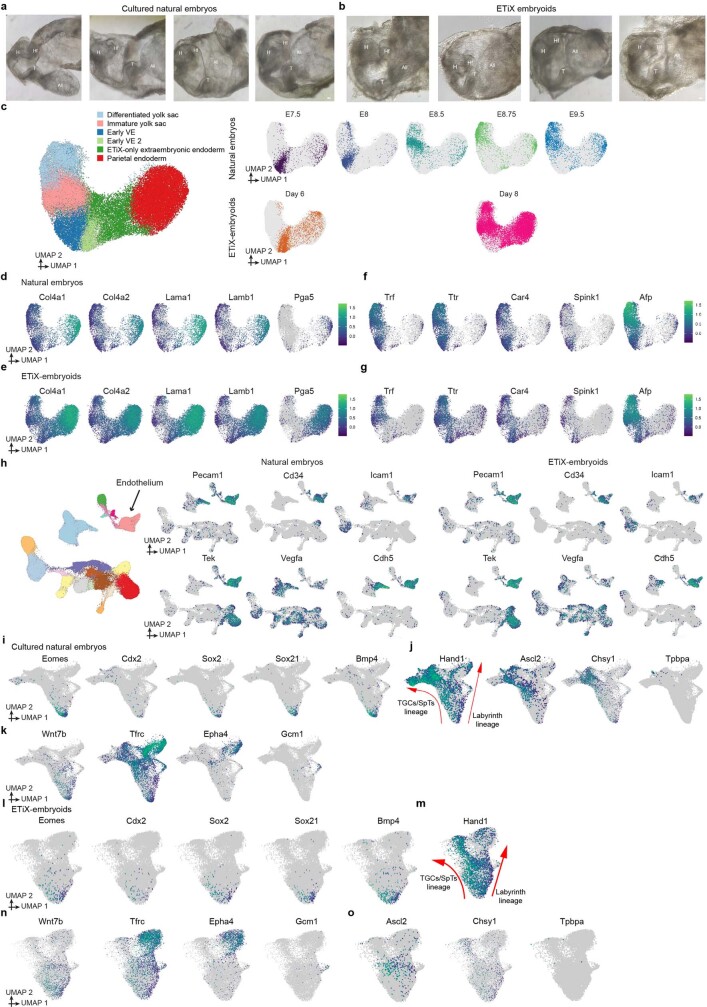
Characterisation of yolk sac, endothelium and extraembryonic ectoderm in ETiX embryoids. **a**,**b**. Partially dissected natural embryos (a) cultured from E6.5 to E8.5 and day 8 ETiX embryoids (b) highlighting their development within extraembryonic membranes. Legend: HF: headfolds, H: heart, T: tailbud and All: allantois. Scale bars, 100 μm **c**. Subclustered UMAP of extraembryonic endoderm in the tiny sci-RNA-seq dataset and contribution of each individual timepoint to the subclustered UMAP of the extraembryonic endoderm. **d**,**e**. Expression of selected parietal endoderm genes in the subclustered UMAP of extraembryonic endoderm shown separately for natural embryos (d) and ETiX embryoids (e). **f**,**g**. Expression of selected extraembryonic visceral endoderm genes in the subclustered UMAP of extraembryonic endoderm shown separately for natural embryos (f) and ETiX embryoids (g). **h**. Expression of selected markers of endothelium *Pecam1*, *Cd34*, *Icam1*, *Tek*, *Vegfa* and *Cdh5* in natural embryos and ETiX embryoids in tiny sci-RNA-seq dataset. **i**. *Eomes*, *Cdx2*, *Sox2*, *Sox21* and *Bmp4* identified a trophoblast progenitor population in natural embryos in the tiny sci-RNA-seq dataset. **j**. Expression of selected markers of the ectoplacental cone lineage. High levels of *Hand1* (left side arrow) show the developmental progression of the ECP lineage towards spongiotrophoblast cells and trophoblast giant cells. Co-expression of *Ascl2* and *Chsy1* indicates committed ECP cells, *Tpbpa* identifies mature spongiotrophoblasts and expression of *Hand1* and prolactin genes (Fig. 5i) denotes the trophoblast giant cells. **k**. Expression of selected markers of chorion progenitors (right side of arrow in *Hand1* UMAP), chorion and differentiated chorion derivatives in natural embryos. *Wnt7b* indicates chorion progenitors, *Tfrc* indicates the chorion cluster, *Epha4* identifies cells of the syncytiotrophoblast layer I and *Gcm1* identifies the syncytiotrophoblast layer II. **l–o** Expression of marker genes presented in (i-k) in the ETiX embryoids UMAP Source data

ETiX embryoids develop inside membranes that resemble the amnion and yolk sac, which provide nourishment to the embryo until the establishment of the fetal–maternal circulation. Both cultured embryos and ETiX embryoids have to be dissected from their yolk sacs (Extended Data Fig. 12a,b) for immunostaining. In both of our sequencing datasets, the amnion and amniotic mesoderm constituted a cluster of cells that expressed the amnion marker *Postn*^52^ (Fig. 6a,b). Additionally, we detected a cluster of cells representative of the allantois tissue marked by the expression of *Tbx4*^53^ and *Hoxa13*^54^ in both cultured natural embryos and ETiX embryoids (Fig. 6c).

During development, the yolk sac originates from cells derived from parietal endoderm and VE, respectively. These populations were both present in our datasets (Fig. 6a). To gain further insight into the diversity of cell types present in these two extraembryonic endoderm lineages, we performed a subclustering analysis (Fig. 6d and Extended Data Fig. 12c). Although the extraembryonic endoderm of ETiX embryoids integrated well with natural embryos (left and far right sides of the UMAP), it also contained cells that were largely absent in natural embryos (ETiX-only extraembryonic endoderm) (Fig. 6d and Extended Data Fig. 12c). These cells had begun to appear in the day 6 embryoids, but the vast majority appeared at day 8 (Extended Data Fig. 12c). In the subclustered UMAP, the parietal endoderm cluster expressed high levels of collagen (*Col4a1* and *Col4a2*) and laminin (*Lama1* and *Lamb1*) genes, as reported previously^17^ and as found in ETiX embryoids at earlier stages^10^ (Extended Data Fig. 12d,e). Consistently, this cluster also expressed the parietal yolk sac marker *Pga5*^54^ (Extended Data Fig. 12d,e). The youngest cluster in the dataset, ‘early VE’ and ‘early VE 2’, expressed genes enriched in the embryonic VE at E7.5 (such as *Spink1*), as well as genes associated with the extraembryonic VE (ExVE) (*Afp*, *Trf*, *Ttr* and *Car4*) (Extended Data Fig. 12f,g). Together this suggests a prevailing ExVE signature, with these ExVE genes being expressed mostly on the left side of the subclustered UMAP (Extended Data Fig. 12c). Gene Ontology analysis of ‘early VE’ clusters and ‘differentiated yolk sac’ clusters highlighted terms consistent with the role of the visceral yolk sac in nutrient transport, lysosome function and lipid and cholesterol metabolism (Supplementary Tables 5 and 6). The ‘immature yolk sac’ cluster was mostly present in ETiX embryoids and lacked a clear expression pattern, suggesting that they might be an aberrant population. The ‘early VE 2’ cluster was present both in natural embryos and in ETiX embryoids and showed expression of the pro-haematopoietic factor *Runx1*, which is known to be restricted to the VE at the boundary of the embryo and ExE^55^. Together, subclustering of the extraembryonic endoderm cells suggested that ETiX embryoids develop parietal yolk sac cells and can acquire mature visceral yolk sac identity, but it also identified cells that are insufficiently mature to undergo the correct developmental programme.

The extraembryonic portion of the yolk sac that developed in ETiX embryoids was attached to a structure resembling the chorion and allantois (Fig. 6e). The yolk sac supports primitive haematopoiesis in the embryo, and notably, we observed RUNX1-positive blood islands in the mesoderm of the yolk sac and at the base of the allantois of neurulating embryoids at day 8 (Fig. 6f). Consistent with the formation of blood islands, genes associated with endothelium^17^ were expressed in the tiny-sci dataset (Extended Data Fig. 12h).

Finally, we sought to characterize the trophoblast compartment in natural embryos to determine whether ETiX embryoids could also develop the cell populations required to form the functional placenta. During development, the proximal portion of the ExE differentiates into the ectoplacental cone (ECP), precursor of trophoblast giant cells and spongiotrophoblasts. The portion of the ExE retaining a stem cell character differentiates into chorion and eventually forms the syncytiotrophoblast layers of the labyrinth, whose surface mediates gas and nutrient exchange between fetus and mother. The ExE subclustered UMAP (Fig. 6g) was annotated using known markers^56^ (Extended Data Fig. 12i–o) and showed the presence of trophoblast precursors, committed and uncommitted ECP, trophoblast giant cells, spongiotrophoblast cells, chorion progenitors, chorion and syncytiotrophoblast cells of layer 1 and 2.

In ETiX embryoids, we observed the continued presence of trophoblast precursors—as in natural embryos—that split into the ECP and chorionic lineages; however, the latter did not perfectly cluster with the chorionic lineages of the natural embryo (Fig. 6g,h, see Extended Data Fig. 12i–o for specific markers). Moreover, the ECP lineage in ETiX embryoids was not fully developed, as expression of ECP genes was altered or absent (ETiX-only ECP) and the lack of prolactin gene expression indicated that trophoblast giant cells and spongiotrophoblast cells were missing (Fig. 6i,j). This analysis showed that whereas development of the chorion lineages had largely taken place, the extraembryonic lineages derived from the ECP were largely absent in ETiX embryoids.

## Discussion

Here we demonstrate the assembly of mouse embryonic and extraembryonic stem cells to form an embryo model that develops the brain, neural tube, heart, foregut, somite, allantois, primordial germ cells and yolk sac structures. This embryo model is able to achieve this entirely through self-organization of these three stem cell types, without the need to provide additional external signalling cues. In contrast to other stem cell-derived embryo models, neurulating embryoids undertake morphogenesis of headfold structures in a manner that closely resembles the natural embryo. This extended development of our embryoids probably relies on their ability to form the anterior signalling centre (the AVE), which protects the anterior embryonic regions from signals that promote posterior development and enables the correct positioning of the primitive streak for gastrulation, as in natural embryos. Together, these events enable the region anterior to the primitive streak to correctly direct formation of fore- and midbrain. The natural gastrulation movements of the ETiX embryoids enable them to proceed to neurulation with formation of the neural tube, initiation of somitogenesis, and the generation of mesodermal structures including a heart-like structure. So far, we have not studied development beyond the establishment of the endodermal progenitors for the gut and its associated organs and it may be necessary to optimize culture conditions to achieve this. However, there are no reasons to suspect that, given appropriate culture conditions, ETiX embryoid development will not proceed further in culture.

Whereas the embryonic lineages of ETiX embryoids capture natural development quite closely, the extraembryonic lineages show some deviation. This might reflect the lack of contact with the maternal environment (natural embryos were recovered when the ECP had already begun developing). Indeed, we and others reported a similar embryo model in which TS cells were exchanged with ES cells induced to express CDX2^57,58^. These studies add to a previous report on the generation of embryoids using ES cells to form the ExE lineage^9^. Embryoids in which the extraembryonic lineages are derived only from ES cells can also develop to neurulation stages. This occurs at lower efficiency than in synthetic embryoids in which only extraembryonic XEN cells are derived from ES cells^57,58^. Our studies show that embryos with induced TS cells show greater deficiencies in the development of their extraembryonic compartment associated with deficiencies in embryonic tissue formation^58^. Thus, although reprogramming of ES cells to the trophectoderm–trophoblast lineage is a valuable way to generate complete embryo models, it requires further optimization.

Notably, we were able to replicate the consequences of *Pax6* knockout in neurulating embryoids, which illustrates the potential of this complete embryo model to dissect the genetic factors that regulate development without the need for experimental animals. We anticipate the widespread application of this system to dissect molecular pathways and to screen for chemical entities that affect embryogenesis. Because ETiX embryoids capture extensive aspects of development, they provide an important opportunity to uncover mechanisms of development and disease.

## Methods

### Cell lines and culture conditions

All cell lines used in this study were mouse cell lines and include the following. CAG-GFP/tetO-mCherry mouse ES cells (constitutive GFP expression in the membrane; transient mCherry expression upon Dox treatment). The parent CAG-GFP/tetO-mCherry ES cell line was derived from an existing mouse line with constitutive CAG-GFP expression and Dox-induced transient mCherry expression. This line was generated by breeding CAG-GFP reporter mice^59^ and tetO-mCherry Histone mice^60^. For the purpose of this study, an independent Dox-inducible Gata4-expressing cassette was introduced into the CAG-GFP/tetO-mCherry ES cell line by *piggyBac*-based transposition, thus mCherry and Gata4 are regulated by two, independent Dox-responsive promoters. CAG-GFP/tetO-mCherry/tetO-Gata4 ES cells generated in-house. Cerl-GFP ES cells (GFP expression under the control of the Cerl-promoter) were derived from a published Cerl-GFP mouse line^61^. Cerl-GFP/tetO-Gata4 ES cells generated in-house. Wild-type CD1 TS cells generated in-house. Wild-type CD1 ES cells (a gift from J. Nichols). CD1/tetO-Gata4 ES cells were generated in-house. Sox2-Venus/Brachyury-mCherry/Oct4-ECFP ES cells (a gift from J. Veenvliet and B. G. Hermann). Blimp1-GFP ES cells (a gift from A. Surani). BVSC ES cells (a gift from W. Reik).

For the experiments reported herein we were able to successfully generate ETiX embryoids with the following ES cell lines: wild-type CD1 ES cells; Sox2-Venus/Brachyury-mCherry/Oct4-ECFP ES cells; CAG-GFP/tetO-mCherry ES cells; Blimp1-GFP ES cells; BVSC ES cells.

In addition to the lines indicated above, we also tried five more that could progress to day 5 and 6 but not beyond. These lines were: Lfng reporter (LuVeLu) ES cells (a gift from A. Aulelha and I. Sonnen); Msgn1-Venus ES cells (a gift from O. Pourquié); Hes7-Achilles ES cells (a gift from O. Pourquié); Sox1-GFP ES cells (a gift from A. Smith); mTmG ES cells (generated by us in-house).

No results reported in this study were generated with these five unsuccessful lines.

The majority of the structures presented in this study were generated using wild-type CD1 ES cells, wild-type CD1 TS cells and CD1/tetO-Gata4 ES cells. The sex of the cell lines is not known because we did not genotype them to determine it. All cell lines were routinely tested every two weeks to ensure that they were not contaminated with mycoplasma. Mouse ES cells and TS cells were cultured as detailed elsewhere^10^. Establishment of CD1 tetO-Gata4 Dox-inducible cell lines was performed as described elsewhere^10^. Cells lines were not authenticated.

### CRISPR–Cas9 *Pax6* knockout

*Pax6* was targeted in the region 104 bp immediately prior to the homeobox sequence on exon 6. guide RNA (gRNA) oligonucleotides were designed using the online CRISPR design tool (www.benchling.com/crispr) and those least likely to have off-targets based on the prediction of the software were selected. gRNAs were annealed with their respective reverse oligonucleotides and cloned into PX459 and transformed into DH5a cells as previously described. Minipreps were sent for Sanger sequencing with sequencing primer: 5′-TGCATATACGATACAAGGCTGTTAG-3′. Wild-type ES cells (CD1 background) were transfected using Lipofectamine 3000 according to the manufacturers’ instructions. In brief, a density of 25,000 cells per well was plated in a 24-well plate the day prior to transfection. The following day, pairs of gRNAs in PX459 were transfected into the cells (500 ng per plasmid). A PIP Fucci construct without an antibiotic resistance cassette in a separate well was used as a control for the transfection. A negative control (no DNA) was also performed in parallel. Following 2 days of selection with 1 μg ml^−1^, the cells were washed in fresh medium and allowed to recover from the antibiotic. Individual clones were isolated and cultured in 96-well plates until colonies became visible. From 46 wells, 18 were growing a single colony. Each of these were passaged and split into 3 new wells, each in a different 96-well plate. Two of these plates were trypsinised and frozen using FC medium + 10% DMSO + 25% FBS. Colonies in the remaining plate were grown until confluency and genomic DNA from each single clone was extracted and genotyped using Platinum Taq DNA Polymerase (Invitrogen, 13001012) and the following primers: FW: 5′-AAGAGACCTTGCGAGAGCAC-3′. RV: 5′-GAACTTTCCCACCAGGAGCA-3′. A standard 25-μl reaction was set up with 12.5 μl Platinum Taq PCR Master Mix, 2 μl template DNA, 0.5 μl of 10μM stock from each primer and 9.5 μl H2O. The PCR cycling conditions in this case were as follows: Initial denaturation was performed at 94 °C for 2 min, followed by 35 cycles of: denaturation at 94 °C for 30 s, annealing at 56 °C for 30 s and extension at 72 °C for 40 s. The PCR product was then examined using gel electrophoresis and promising clones that ran at sizes lower than the wild-type (lower than 715 bp) were sent for Sanger sequencing. Deletion was confirmed by immunofluorescence following a neural differentiation protocol^62^.

### Formation of ETiX embryoids

A step-by-step protocol is available on Protocol Exchange^63^

Formation of ETiX embryoids was performed as previously described^10^. In brief, cells were plated in the AggreWell (day 0). On the next day (day 1), medium change was performed twice by removing 1 ml of medium from each well and adding 1 ml of fresh FC medium without ROCK inhibitor. On day 2, medium change was performed once to replace 1 ml of medium with 1 ml of fresh FC medium. On day 3, 1 ml of medium was removed from each well and 1.5 ml of IVC1 (with FBS at 20% v/v)^64^ was added, after equilibrating for 20 min in the incubator. On day 4, ETiX embryoids in the AggreWell were transferred to Cellstar 6-well multiwell plate for suspension culture (Greiner Bio-One 657185) with 5 ml of IVC1 (with FBS at 30% v/v) per well.

Ex utero culture of mouse embryos was as described^12,13^. DRH medium^12^ comprises 25% DMEM, 50% rat serum and 25% human cord serum, and permits development to the somite stage in stationary culture and beyond following transfer to the Precision rotating bottle culture apparatus (BTC Engineering)^12^. Glutamine and antibiotics (100 units ml^−1^ penicillin and 100 μg ml^−1^ streptomycin) were added to bicarbonate-buffered DMEM without glutamine (Gibco 11054). As depletion of glucose has been described to be a major cause of malformations and growth retardation^12,13^, the low glucose DMEM (1 mg ml^−1^) was supplemented with 3 mg ml^−1^ glucose. As phenol red is fluorescent, we routinely culture embryos in medium lacking phenol red. The medium of Aguilera-Castrejon et al.^13^ had the same proportions of DMEM (Gibco 11880), rat serum and human cord serum as DRH, but was buffered with HEPES rather than bicarbonate (and was renamed EUCM medium). Rat whole embryo culture serum was from Charles River and human cord serum was provided by the Cambridge Blood and Stem Cell Biobank, which is supported by the Cambridge NIHR Biomedical Research Centre, Wellcome Trust—MRC Stem Cell Institute and the Cambridge Experimental Cancer Medicine Centre, UK. Human and rat serum were heat-inactivated for 35 min (from frozen) at 56 °C and sterilized by filtration^13^.

Routinely, on day 5, we replaced IVC1 with DRH or EUCM containing 1× Glutamax (GIBCO, 35050061), 100 units ml^−1^ penicillin and 100 μg ml^−1^ streptomycin and 11 mM HEPES (GIBCO 15630056). Each ETiX embryoid was transferred to a single well of 24-well, non-adherent dish (Greiner 662102) with 250 μl DRH or EUCM. On day 6 each ETiX embryoid was fed with an additional 250 μl of DRH or EUCM. A supplement of 3.0 mg ml^−1^ (Sigma G8644) was added to low glucose medium on day 7 when the samples were moved to the rotating bottle culture chamber apparatus. Each rotating bottle contained 2 ml medium and 3 ETiX embryoids. On day 8 the medium was further supplemented with 3.5 mg ml^−1^ d-glucose. In each rotating bottle, 2 ETiX embryoids were cultured with 3 ml medium.

A device to regulate gas pressure and gas mixing on the roller bottles as described^13^ was provided by J. Hanna and A. Aguilera-Casterjon. The device was modified to enable pressure generation but not gas mixing, and we therefore used only the pressure-generating component of this device to enable development of embryoids to day 8. We also built our own device that delivers defined gas mixtures but at 0.5 psi rather than 6.5 psi, as described^13^. We used this device to successfully culture embryoids and natural embryos for sequential smFISH. We found that delivery of gas mixtures at this lower pressure was effective in promoting development either when 21% oxygen was continuously supplied during culture or when the oxygen concentration was incrementally increased from 5% to 13% to 18% to 21% at daily intervals. DRH medium necessitates use of 5% CO_2_.

### Mouse model and embryo recovery

Mice (six-week-old CD-1 males from Charles River and transgenic females bred in house) used in the experiments were kept in animal house, following national and international guidelines. All experiments performed were under the regulation of the Animals (Scientific Procedures) Act 1986 Amendment Regulations 2012 and were reviewed by the University of Cambridge Animal Welfare and Ethical Review Body (AWERB). Experiments were also approved by the Home Office. Mice were maintained in the animal facility at 12:12 light cycle and provided with food and water ad libitum.

Natural mating was performed with six-week-old transgenic females and CD-1 males. Mouse embryos were recovered at embryonic days E5.5, E6.5 and E7.5 by dissecting them from the deciduae in M2 medium (Sigma M7167). Embryos at E6.5 were cultured in EUCM in stationary conditions until E8.5 as reported^13^ in the same way as the ETiX embryoids. At E8.5, embryos were moved to a rotating bottle in DRH or EUCM supplemented with 3.0 mg ml^−1^ of d-glucose. Each bottle contained 2 ml of DRH or EUCM medium and 3 embryos. Both male and female embryos were used. Embryos were randomly allocated and researchers were not blinded for embryo allocation.

### Immunofluorescence

ETiX embryoids and natural embryos were processed for immunofluorescence as previously reported^10^. Natural embryos older than E7.5 and ETiX embryoids older than day 6 were permeabilized for 35 min.

### Cryosectioning and slide immunofluorescence

Natural embryos and ETiX embryoids samples after fixation were cryoprotected in 30% sucrose/PBS (w/v) overnight at 4 °C. Samples were then transferred to a cryomold filled with OCT compound (Agar Scientific) and frozen on a metal block cooled on dry ice. The samples were cut at a thickness of 12 μm on a cryostat, collected on lysine coated slides and stored at −80 °C until ready for immunofluorescence. Slides were washed in PBS to remove OCT for 5–10 min and then briefly allowed to air dry for 5–10 min. Samples were permeabilised for 10 min at room temperature with permeabilization buffer (0.1 M glycine and 0.3% Triton X-100 in PBST) and then blocked for 1 h at room temperature with blocking buffer (10% FBS and 0.1% Tween 20 in PBS). After permeabilization and blocking, samples were processed as described above. Slides were mounted with Vectashield, sealed with nail polish, and allowed to air dry overnight in the dark prior to imaging.

### inDrops scRNA-seq sample preparation and dissociation

After recovery, natural embryos and ETiX embryoids were dissociated for single-cell sequencing as previously described^10^. Samples analysed: *n* = 29 for ETiX5, 10 for ETiX6, 7 for ETiX8, 12 for E6.5, 14 for E7.5 and 9 for E8.5.

### inDrops scRNA-seq library preparation and sequencing

Libraries were prepared according the inDrops v3^14^ workflow^15^ with v3 barcoding scheme^16^. In brief, polyacrylamide beads were generated and barcoded to obtain a diversity of 147,456 barcodes. Single-cell suspensions were diluted to a concentration of 100,000 cells per ml and co-encapsulated with the barcoded beads and reverse transcriptase and lysis mix. Fractions of ~1,000 cells were collected in 1.5 ml Eppendorf tubes pre-filled with 200 µl mineral oil, subjected to UV photocleavage and incubated at 50 °C for 2 h and 70 °C for 20 min. The droplets were then de-emulsified and further amplified using second-strand synthesis and in vitro transcription. The libraries were then fragmented and reverse transcribed. The final libraries were amplified using a unique 8-bp index using limited-cycle PCR and quantified using a Qubit High sensitivity kit (Invitrogen) and Bioanalyzer High sensitivity DNA kit (Agilent). Libraries were pooled at equi-molar ratios and purified using a 1.5× volumetric ratio of AmpureXP beads. The libraries were sequenced on a Nextseq 75 cycle 400M read High Output kit with 5% PhiX spike-in as an internal control. The read cycle distribution was the following: read 1, 61 cycles; index 1, 8 cycles; index 2, 8 cycles; read 2, 14 cycles.

### scRNA-seq bioinformatic analysis

The BCL files were converted to Fastq files using Illumina’s bcl2fastq software. The sequenced libraries were quality-inspected using the FastQC tool v0.11.9 (https://www.bioinformatics.babraham.ac.uk/projects/fastqc/) and de-multiplexed using the Pheniqs tool from biosails v2.1.0. The fastq files were further filtered, mapped to a mouse GRCm38.99 reference genome with GRCm38.99 gtf annotation and deduplicated using the zUMIs pipeline^65^ v2.9.7. The count matrices with exonic and intronic counts were then used as an input for downstream analysis using Seurat^66^ version 3. Cells were filtered based on the number of genes detected (between 700 and 4,000), unique molecular identifiers (UMIs) detected (lower than 7,500), percentage of UMI counts mapping to mitochondrial genes (between 1 and 15%) and doublet scores computed using Scrublet^67^ v0.1 (lower than 0.3), which yielded a total of 26,748 cells overall. The natural and synthetic embryo datasets were integrated in Seurat and shared embeddings were corrected for batch effect (both systems and timepoints collected) using Harmony^68^ v4.3.12. Louvain clustering was performed on the shared embeddings and markers, computed using the FindAllMarkers function from Seurat, were used to annotate cell types. Pearson correlation coefficients between cell types for each system, single-cell velocity profiles and latent times were computed using the Scanpy^69^ v1.0 and scVelo^70^ v0.2.4 tools. Plots were generated using Scanpy (in Python for dot plots and velocity) and Seurat (in R for UMAP plots), as well ggplot2 for the remainder of the plots (in R for bar plots and proportion scatter plots).

### Generating single-cell sequencing data using tiny-sci-RNA-seq

We performed a simplified version of sci-RNA-seq3, further optimized for ‘tiny’ samples^20^. In brief, to each tube, 100 μl of a hypotonic, PBS-based lysis buffer was added with DEPC as an RNase inhibitor. The resulting nuclei were then fixed with four volumes of a mix of methanol and dithiobis (succinimidyl propionate) (DSP). After rehydrating and washing the nuclei carefully in a sucrose/PBS/triton/MgCl_2_ buffer (SPBSTM), the nuclei were distributed to two 96-well plates for reverse transcription, allocating 8 wells per embryo. After reverse transcription, nuclei were pooled, washed in SPBSTM and redistributed to a fresh plate for ligation of the second index primer with T4 DNA ligase. Nuclei were then again pooled, washed, and redistributed to three final plates for second-strand synthesis, extraction, tagmentation, and PCR to add the third index plus a plate index. Products were pooled by PCR plate, size-selected and sequenced on two Illumina NextSeq runs (NextSeq-1 and NextSeq-2). Samples analysed: *n* = 8 natural embryos ranging from E7.5 to E9.5, *n* = 3 for ETiX6, *n* = 2 for failed ETiX6, 5 for ETiX8, 4 failed ETiX8 and 2 *Pax6-*knockout ETiX8.

### Processing of sequencing reads of tiny-sci-RNA-seq data

For each NextSeq run of newly generated tiny-sci-RNA-seq data, read alignment and gene count matrix generation was performed using the pipeline that we developed for sci-RNA-seq3^71^ with minor modifications: base calls were converted to fastq format using Illumina’s bcl2fastq v2.20 and de-multiplexed based on PCR i5 and i7 barcodes using maximum likelihood demultiplexing package deML^72^ with default settings. Downstream sequence processing and single-cell digital expression matrix generation were similar to sci-RNA-seq^73^ except that reverse transcription (RT) index was combined with hairpin adaptor index, and thus the mapped reads were split into constituent cellular indices by demultiplexing reads using both the RT index and ligation index (Levenshtein edit distance (ED) < 2, including insertions and deletions). In brief, de-multiplexed reads were filtered based on RT index and ligation index (ED < 2, including insertions and deletions) and adaptor-clipped using trim_galore v0.6.5 with default settings. Trimmed reads were mapped to the mouse reference genome (mm10) for mouse embryo nuclei, using STAR v2.6.1d^74^ with default settings and gene annotations (Gencode VM12 for mouse). Uniquely mapping reads were extracted, and duplicates were removed using the UMI sequence (ED < 2, including insertions and deletions), RT index, hairpin ligation adaptor index and read 2 end-coordinate (that is, reads with UMI sequence less than 2 edit distance, RT index, ligation adaptor index and tagmentation site were considered duplicates). Finally, mapped reads were split into constituent cellular indices by further demultiplexing reads using the RT index and ligation hairpin (ED < 2, including insertions and deletions). To generate digital expression matrices, we calculated the number of strand-specific UMIs for each cell mapping to the exonic and intronic regions of each gene with Python v2.7.13 HTseq package^75^. For multi-mapped reads, reads were assigned to the closest gene, except in cases where another intersected gene fell within 100 bp of the end of the closest gene, in which case the read was discarded. For most analyses we included both expected-strand intronic and exonic UMIs in per gene single-cell expression matrices. After the single-cell gene count matrix was generated, doublets cells and potential low quality cells (by investigating the numbers of UMIs and the proportion of reads mapping to the exonic regions per cell) were filtered out and 285,640 cells were left (*n* = 130,611 cells for NextSeq-1, and *n* = 155,029 cells for NextSeq-2; Extended Data Fig. 3a–d). The following common, freely available data analysis software was used in this project: scrublet version 0.1 (https://github.com/swolock/scrublet), Scanpy version 1.6.0 (https://github.com/theislab/scanpy), Monocle versions 2, 3 and 3-alpha (https://cole-trapnell-lab.github.io/monocle3), Seurat version 3 (https://github.com/satijalab/seurat) and ggplot2 version 3.3.5 (https://ggplot2.tidyverse.org/).

#### Subclustering

We performed conventional scRNA-seq data processing using Seurat v3: (1) normalizing the UMI counts by the total count per cell followed by log transformation; (2) selecting the 2,500 most highly variable genes and scaling the expression of each to zero mean and unit variance; (3) applying principal component analysis and then using the top 30 principal components to create a *k*-nearest neighbours graph, followed by Louvain clustering (resolution = 1); (4) performing UMAP visualization in 2D space (dims (which dimensions to use as input features) = 1:30, min_dist = 0.3). We manually merged neighbouring clusters if there were limited number of differential expressed genes between them. For subclustering, we took a subset of cells of interest (for example, cardiac mesoderm) and followed the above approach to identify more detailed cell populations.

### Identification of correlated cell types between ETiX embryoids and natural embryos using NNLS regression

We first aggregated transcriptional profiles for cells within each cell type for either ETiX embryoids or natural embryos in the newly generated tiny-sci-RNA-seq data. We applied NNLS regression to predict gene expression in target cell type (*T*_*a*_) in dataset A based on the gene expression of all cell types (*M*_*b*_) in dataset B: *T*_*a*_ *= β*_*0a*_ *+ β*_*1a*_*M*_*b*_ (where *β*_0a_ is the intercept of the regression and *β*_1a_ is the *β*-coefficient of the regression), based on the union of the 3,000 most highly expressed genes and 3,000 most highly specific genes in the target cell type. We then switched the roles of datasets A and B—that is, predicting the gene expression of target cell type (*T*_*b*_) in dataset B from the gene expression of all cell types (*M*_*a*_) in dataset A: *T*_*b*_ *= β*_*0b*_ *+ β*_*1b*_*M*_*a*_. Finally, for each cell type *a* in dataset A and each cell type *b* in dataset B, we combined the two correlation coefficients: *β* = 2(*β*_*ab*_ + 0.01)(*β*_*ba*_ + 0.01) to obtain a statistic for which high values reflect reciprocal, specific predictivity.

### Inclusion criteria of ETiX embryoids

All ETiX embryoids were collected from AggreWell for analysis at 4 days of development and analysed under a stereomicroscope and we selected ETiX embryoids with cylindrical morphology and two clearly defined cellular compartments (an ES cell compartment and TS cell compartment) surrounded by an outer cell layer, the VE-like layer. We expect the ES cell compartment to be epithelialized with a lumen. The TS cell compartment is more variable in appearance and therefore, even though one would also want an epithelial-looking TS cell compartment similar to the ExE of natural embryos, we select a wider range of appearances for the TS cell compartment. Since the majority of ETiX embryoids were generated by using wild-type, unlabelled stem cell lines, the selection was based on morphology alone. ETiX embryoids with the correct body plan of ES cell and TS cell compartments surrounded by a VE-like layer were then transferred to equilibrated medium to continue their culture. When selecting at day 5, however, we included additional criteria: (1) we expect the lumen of the ES cell and TS cell compartment to be merged; (2) ideally, we can observe the beginning of gastrulation on one side of the ETiX embryoids; (3) we expect the AVE to have migrated to the ES cell–TS cell boundary and be opposite to the forming streak; (4) ETiX embryoids with the AVE stuck at the tip of the structure or not at the boundary were excluded. At day 4, we were able to collect 10%–15% of the structures formed in the pyramidal microwells. From day 4 to day 5 we cultured 20% of the structures collected at day 4.

### Image acquisition, processing and analysis

Images were acquired using Leica SP5 and SP8 confocal microscopes (Leica Microsystems) with 40× oil objective and 25× water objective, respectively. A 405-nm diode laser (DAPI), a 488-nm argon laser (Alexa Fluor 488), a 543-nm HeNe laser (Alexa Fluor 568) and a 633-nm HeNe laser (Alexa Fluor 647) were used to excite the fluorophores. Images were taken with a *z*-step of 1.2–5 µm. FIJI^76^ and NDSAFIR 3.0^77^, the Smart Denoise (Gurdon Institute) were used to process and analyse the images. Area measurements used to generate the quantifications were also collected using Fiji. Figures were assembled with Adobe Illustrator v26.0.1.

The images we provide are representative of multiple experiments analysed. As with natural embryos, the features of the ETiX embryoids described can be observed under a regular stereomicroscope—for example, the beating heart-like structure is clearly visible in 87% of the structures we obtain, and the headfolds and somites are readily observable and recognizable. Observation of such morphology in bright-field microscopy is predictive of staining reproducibility. Furthermore, analysis by single-cell sequencing of individual ETiX embryoids shows great reproducibility among these structures, and our quantifications suggest that, albeit greater size variability is observed across ETiX embryoids, the organs and regions analysed are of comparable size to those of natural embryos. Finally, it is also likely that some of the variability we observe in ETiX embryoids is caused by ex utero culture itself, since it causes variability in the development of natural embryos as well.

### Sequential smFISH

#### Primary probe design

Gene-specific primary probe sets were designed as previously described with some modifications^78^. In brief, probe sets of 35-nt binding sites were crafted for each gene using exonic sequences from the consensus regions for all spliced isoforms. For genes that did not yield enough targets (>40), the intronic and 5′ untranslated region sequences were also used. The masked genome and annotation databases from UCSC were used to obtain the gene sequences and extract 35-bp sequences with 45–75% GC content and without a region of repeating 5-nt bases of the same kind. Each probe sequence was then run against a BLAST database constructed from GENCODE-reversed introns and mRNA sequences. All probes with BLAST hits on any sequence other than the target gene with at least a 15-nt match were considered off-target hits and dropped from the probe set. All probe sets for each gene were then trimmed to a maximum of 40 probes, removing all probes that lie furthest from the targeted 55% GC content.

#### Readout probe design

Readout probes were used as previously designed^78^. In brief, a set of 20-nt probe sequences was generated randomly by combinations of A, T, G and C nucleotides. Sequences of 45–60% GC were selected and run against a BLAST database to eliminate any sequences that matched with any contiguous homology sequences longer than 14 nt to the mouse transcriptome. The reverse complements of these readout sequences were included in the primary probes with AA or TAAT linkers in a manner as follows: [readout]-AA-[readout]-AA-[readout]-TAAT-[probe binding sequence]-TAAT-[readout]-AA-[readout]-AA-[readout]. Thus, each probe was 141 nt long.

#### Primary probe and readout probe construction

Primary probes were ordered as IDT oligonucleotide pools with 50 pmol per probe concentration and 5′-phosphate modification. Readout probes were ordered from IDT as 250 nmol DNA oligonucleotides with HPLC purification and 5′-fluorophore modifications (5′ Alexa Fluor 647N, 5′ Alexa Fluor 488N or 5′ Alexa Fluor 546N).

#### Coverslip functionalization

Coverslips were functionalized by 1 M HCl treatment at room temperature for 1 h, rinsed with water once, 1 M NaOH treatment at room temperature for 1 h, then immersion in 1% bind-silane (GE-Healthcare, 17-1330-01) prepared in pH 3.5 10% (v/v) acidic ethanol solution for 30 min. The coverslips were then thoroughly rinsed in 100% ethanol 3 times and placed on glass slides for heat-curing in an oven at >90 °C for 30 min. The coverslips were allowed to cool down and the area of the coverslip intended for final tissue section placement was covered in 100 µg µl^−1^ of poly-d-lysine (Gibco, A3890401) for >1 h. The coverslips were then thoroughly rinsed in water three times and then allowed to dry in a tissue culture hood with UV-sanitation. For long-term storage, the coverslips were kept dry at 4 °C for <2 weeks before use.

#### Sequential smFISH experiment on ETiX embryoids

ETiX embryoids and natural embryos were fixed with 4% paraformaldehyde at 4 °C overnight. They were then washed with PBST (PBS with 0.1% Tween 20) twice at 4 °C and dehydrated into methanol gradually with a series of graded methanol/PBST washes for 10 min each at 4 °C. Samples were stored at −20 °C overnight before they were rehydrated in a series of graded methanol/PBST washes and washed twice in PBST at 4 °C for 10 min. Embryo samples were then immersed in 30% sucrose/PBS (w/v) overnight at 4C or until the sample sank to the bottom of the tube. Samples were transferred to a cryomold, carefully positioned in optimal cutting temperature (OCT) compound solution (Agar Scientific), and frozen in dry ice ethanol. Samples were stored at −80 °C before sectioning.

The tissue blocks were cut at 20-µm thickness using a micron cryostat and placed onto the functionalized coverslips. After drying out for >15 min, the coverslips were placed at −80 °C for long-term storage. After at least one day of storage at −20 °C, the tissue section was permeabilized in 70% ethanol at 4 °C for >1 h, then dried and cleared with 1 ml of 8% SDS (Invitrogen, AM9822) in 1× PBS at room temperature for 30 min. After rinsing with PBS two times and nuclease-free water one time, a custom-made flow cell (fluidic volume about 30 µl), which were made from glass slide (25 × 75 mm) with 1 mm thickness and 1 mm diameter holes and a PET film coated on both sides with an acrylic adhesive with total thickness 0.25 mm (Grace Bio-Labs, RD481902) was attached to the coverslips. Using the 1 mm diameter holes, the tissue sample was incubated in 30% hybridization buffer (Molecular Instruments) containing 3.3 nM of each probe overnight at 37 °C. The holes of the flow cell were covered using a sticker (Grace Bio-Labs, GBL629200) to prevent evaporation during incubation. The sample was then washed with 30% wash buffer (Molecular Instruments) for 4 times over 1 h, then rinsed with 4× SSC (Thermo Fisher, 15557036) 5 times.

#### Sequential smFISH on control embryos

All steps for ETiX embryoid smFISH experiment were followed with the additional clearing steps to improve signal-to-noise ratio in the resulting images. In addition to the gene marker probes, a poly-30-T LNA oligonucleotide with 5′-acrydite modification (IDT) was hybridized at 2 µM. After 30% wash buffer washes, a sticker containing a circle cutout of 3 mm diameter and 100 µm thickness was applied to the sample. A gel solution containing 4% acrylamide and 0.2% bis-acrylamide (Bio-Rad, 1610154) with 0.25% VA-044 (Fujifilm, LB-VA044-50GS) was assembled on ice and treated with nitrogen for >5 min. Twenty microlitres of the solution was dropped onto the sample (contained inside the circle cutout within the sticker) and a 22 × 22 mm coverslip was applied on top of the sample. The sample was then placed into a humidified airtight chamber and all the oxygen was removed by flowing in nitrogen for >10 min. To allow infiltration of the sample with the hydrogel, the sample was incubated at 4 °C overnight, then placed at 37 °C for 3.5 h to allow the hydrogel to solidify. The small glass coverslip and sticker was then removed, and the sample was treated with 1:100 Proteinase K (NEB, P8107S), 50 mM pH 8.0 Tris-HCl (Thermo Fisher, 15568025), 1 mM EDTA (Thermo Fisher, 15575020), 0.5% Triton X-100 (Sigma, 93443), 500 mM NaCl (Sigma, S5150), and 1% SDS (Invitrogen, AM9822) for 2.5 h at 37 °C in a humidified chamber. The sample was then washed with 2× SSC for 15 min, then treated with Label-X as previously described (0.1 µg µl^−1^ of Acryoyl-X SE (Thermo Fisher A20770) and 0.1 µg µl^−1^ of Label-IT Reagent (Mirus, MIR3900)) for 45 min at 37 °C. The sample was washed with 2× SSC, then re-embedded in a hydrogel as done above for further stabilization for long-term imaging.

#### Microscope setup

All imaging experiments were performed with the imaging platform and fluidics delivery system like those previously described^78^. The microscope (Ti Eclipse) was equipped with a confocal scanner unit (Yokogawa CSU-W1), a sCMOS camera (Andor Zyla 4.2), a 60× oil objective (Nikon Plan/Apo, NA 1.4, WD 0.13), a motorized stage (ASI MS-2000), and a 7-wavelength Nikon LUNF XL laser launch. The following filters were used: 435/26 bp (Chroma) for 405 nm, 525/36 bp (Chroma) for 488 nm, 588–700 bp (Chroma 59007 dual band pass) for 561 nm, and 705/72 bp (Chroma) for 647 nm. A custom-made automated sampler was used to move designated readout probes in hybridization buffer (10 nM per readout probe in 2× SSC, 10% ethylene carbonate (Sigma, E26258), 10% dextran sulfate (Sigma, D4911), and 0.1 µg/mL DAPI (Thermo Fisher, D1306)) from a 2.0 ml 96-well plate through a multichannel fluidic valve (IDEX Health and Science, EZ1213-820-4) to the custom-made flow cell using a syringe pump (Hamilton Company, 63133-01). Other buffers, like 2× SSC, 10% wash buffer (2× SSC, 10% formamide (Thermo Fisher, AM9342), 0.1% Triton X-100 in nuclease-free water), 55% wash buffer (2× SSC, 55% formamide, 0.1% Triton X-100 in nuclease-free water), and anti-bleaching buffer (3 mM Trolox (Sigma, 238813), 1% w/v d-glucose (Sigma, G7528), 1:100 diluted catalase (Sigma, C3155), 1.0 mg ml^−1^ glucose oxidase (Sigma, G2133), and 50 mM pH 8.0 Tris-HCl) were also moved through the multichannel fluidic valve to the custom-made flow cell using the syringe pump. The integration of imaging and the automated fluidics delivery system was controlled by custom-written scripts in µManager and Python using Jupyter notebooks.

#### Imaging

The sequential hybridization and imaging routines were performed similarly to those previously described^78^ with some modifications. The sample with the custom-made flow cell was first connected to the automated fluidics system on the motorized stage on the microscope. The region of interest (8 × 8 tile scan with 25% overlap for ETiX embryoid, 8 × 9 tile scan with 20% overlap for control embryo) was identified and used for sequential rounds of hybridization and imaging as follows. The hybridization buffer with readouts and DAPI was flowed onto the sample and allowed to incubate for 60 min, then washed with 10% wash buffer for 1 min, and washed with 2× SSC three times before applying anti-bleaching buffer for image acquisition. *Z*-stacks with sections of optical thickness of 0.65 µm were acquired for each tile, using 500–2,000 ms exposure times for all lasers used. After image acquisition, the readouts were washed off the sample with 3 washes of 55% wash buffer, with 5-min incubation periods between each wash, followed by one wash with 2× SSC. This process was then repeated for all rounds of gene markers. For the last round of smFISH imaging, the sample was stripped of readout probes and incubated with 0.1 µg ml^−1^ DAPI in 2× SSC for 30 min prior to imaging in all channels.

Each readout probe hybridization and stripping routine took approximately 1.5 h. Imaging time per tile took around 8 min each, so that the entire tile scan took around 8.5 h. Therefore, it took approximately 5 days to complete the 11 rounds of hybridization and imaging routine for each experiment.

#### Image analysis

To increase signal-to-noise ratio in the ETiX embryoid images, the last round of imaging (DAPI only, without readout probes) was used to subtract away the sample background signal. This was not done for the control embryo FISH images because the background in that experiment was minimal due to the additional hydrogel clearing steps. Thus, for the ETiX embryoid images, each tile of the background round was registered using the phase_cross_correlation function (without normalization) from the skimage package in Python to the corresponding tile of each smFISH round using the DAPI channel as reference to generate a shift vector. The shift vector was then applied to the rest of the channels (488, 561 and 647 nm) for the background round and the registered sample background was subtracted against the corresponding channel for each tile of each smFISH ETiX embryoid round.

For both the ETiX embryoid and control embryo FISH images, the background signal was further reduced for each tile by using the ImageJ rolling ball background subtraction algorithm with a radius of 50 pixels. Each DAPI tile was then max-projected and the tiles of each round were then stitched together using the ImageJ Grid/Collection stitching algorithm with regression threshold as 0.01, without overlap computation, and standard settings otherwise. To stitch the other channels in the same manner as the DAPI channel, the rest of the channels were then max-projected and the tiles for each round were stitched together using the settings acquired from the tile configuration file generated from the DAPI tile stitching.

An Ilastik classifier was then trained and used to classify the signal for each channel (405, 488, 561 and 647 nm) of each FISH round into foreground and background. The DAPI foreground mask was dilated using a disk structuring element with 10 pixel radius and used to mask out any erroneous foreground signal from the 488, 561 and 647 nm channel that lay far outside of nuclei-positive imaging regions. The foreground for each of the 488, 561 and 647 nm channels was distance transformed and used to find local minima, which were used to perform a watershed to obtain a final label image in which each label represents one detected, unique transcript. To visualize gene expression across different imaging rounds, the stitched DAPI images were then registered across different rounds to obtain a shift vector for each round. This shift vector was then applied to the label positions corresponding to their respective round. For figure visualization, the centre of each label was then plotted as a disk with 7-pixel radius.

### Gene Ontology analysis

Gene Ontology for the extraembryonic endoderm was performed using the online platform DAVID^79,80^. Differentially expressed genes from pairwise comparisons were selected by choosing genes with an adjusted p-value < 0.05 and enriched in one sample or the other of the pariwise comparison. The list was then uploaded in the DAVID user interface and analysed with the Gene Functional Annotation Clustering tool and the Gene Functional Annotation Table. The first 20 clusters with the highest Enrichment Score (−log P*-*value) were graphed. Gene Ontology analysis was performed using to explore the functional roles of differentially expressed mRNAs in terms of ‘biological processes’ between natural embryos and ETiX embryoids, and between wild-type and *Pax6*-knockout ETiX embryoids using the GO Enrichment Analysis online tool (GO Ontology database, 10.5281/zenodo.6399963, released on 22 March 2022)^81–83^. Differentially expressed genes for the pairwise comparison were selected as described above. GO terms were inputted into excel for graphing purposes.

### Statistical analysis

Data were tested for normality using the Shapiro–Wilk test. Normally distributed data was analysed with unpaired *t*-test as indicated in the figure legends. Data that was not normally distributed was analysed with a Mann–Whitney *U*-test. All tests were performed in Prism GraphPad software v9.2. Number of samples for the statistical test is indicated in the figure legends. All tests were performed as two-tailed. For each test, different samples were used with the exception of Fig. 4f, in which multiple somites per sample were measured to determine the area and with the exception of Extended Data Fig. 7i, in which multiple sections of a heart sample were measured for area measurements. All replicates were biological, not technical. Sample size was not predetermined. Sample allocation was randomized. Researchers were not blinded to the type of samples that they were working with.

### Antibodies

A list of antibodies used in this study can be found in Supplementary Table 7.

### Oligonucleotides

A list of oligonucleotides used in this study can be found in Supplementary Table 8.

### Reporting summary

Further information on research design is available in the Nature Research Reporting Summary linked to this article.

## Online content

Any methods, additional references, Nature Research reporting summaries, source data, extended data, supplementary information, acknowledgements, peer review information; details of author contributions and competing interests; and statements of data and code availability are available at 10.1038/s41586-022-05246-3.

### Supplementary information

Reporting Summary

Supplementary Table 1 **Gene Ontology of E7.5 embryos**. Gene ontology analysis of the differentially expressed genes enriched in natural embryos cultured from E6.5 to E7.5 ex utero and analysed by tiny-sci.

Supplementary Table 2 **Gene Ontology of ETiX6 embryoids**. Gene ontology analysis of the differentially expressed genes enriched in ETiX embryoids analysed at day 6 of development by tiny-sci.

Supplementary Table 3 **Gene Ontology of E8.5 embryos**. Gene ontology analysis of the differentially expressed genes enriched in natural embryos cultured from E6.5 to E8.5 ex utero and analysed by tiny-sci.

Supplementary Table 4 **Gene Ontology of ETiX8 embryoids**. Gene ontology analysis of the differentially expressed genes enriched in ETiX embryoids analysed at day 8 of development by tiny-sci.

Supplementary Table 5 **Gene Ontology of Extraembryonic endoderm**. Gene Ontology analysis of the genes enriched in ‘early VE’ cell populations in the extraembryonic endoderm subcluster of ETiX embryoids.

Supplementary Table 6 **Gene Ontology of older cell clusters in Extraembryonic endoderm**. Gene Ontology analysis of the genes enriched in ‘differentiated yolk sac’ cell populations of ETiX embryoids.

Supplementary Table 7 **Antibody Table**. A table of the antibodies utilised in this study.

Supplementary Table 8 **Oligonucleotide Table**. A table of the oligonucleotides utilised in this study.

Supplementary Video 1 **Live imaging of ETiX8 embryoid showing beating heart**. *n* = 11 structures from 4 independent experiments.

Supplementary Video 2 **Live imaging of ETiX8 embryoid showing beating heart**. *n* = 11 structures from 4 independent experiments.

### Source data

Source Data Fig. 1

Source Data Fig. 2

Source Data Fig. 3

Source Data Fig. 4

Source Data Fig. 5

Source Data Fig. 6

Source Data Extended Data Fig. 1

Source Data Extended Data Fig. 3

Source Data Extended Data Fig. 4

Source Data Extended Data Fig. 5

Source Data Extended Data Fig. 6

Source Data Extended Data Fig. 7

Source Data Extended Data Fig. 8

Source Data Extended Data Fig. 10

Source Data Extended Data Fig. 12

## Data Availability

All unique and stable reagents generated in this study are available from the corresponding author with a completed Materials Transfer Agreement. Raw single-cell sequencing data generated by this work have been deposited in the NCBI Gene Expression Omnibus database (https://www.ncbi.nlm.nih.gov/geo/) and are accessible through the following accession numbers: the inDrops scRNA-seq dataset is available at GSE189425; the tiny-sci-RNA-seq dataset is available at GSE209792. Source data are provided with this paper.

## Code availability

The code used to analyse the inDrops scRNA-seq data is available at https://github.com/fhlab/scRNAseq_inducedETX The code used to analyse the tiny-sci-RNA-seq data is available at https://github.com/ChengxiangQiu/ETiX_Amadei.

## References

[1] ten Berge D (2008). Wnt signaling mediates self-organization and axis formation in embryoid bodies. Cell Stem Cell.

[2] van den Brink SC (2014). Symmetry breaking, germ layer specification and axial organisation in aggregates of mouse embryonic stem cells. Development.

[3] Xu P-F (2021). Construction of a mammalian embryo model from stem cells organized by a morphogen signalling centre. Nat. Commun..

[4] Beccari L (2018). Multi-axial self-organization properties of mouse embryonic stem cells into gastruloids. Nature.

[5] Veenvliet J (2020). Mouse embryonic stem cells self-organize into trunk-like structures with neural tube and somites. Science.

[6] Harrison SE, Sozen B, Christodoulou N, Kyprianou C, Zernicka-Goetz M (2017). Assembly of embryonic and extra-embryonic stem cells to mimic embryogenesis in vitro. Science.

[7] Sozen B (2018). Self-assembly of embryonic and two extra-embryonic stem cell types into gastrulating embryo-like structures. Nat. Cell Biol..

[8] Girgin MU (2021). Bioengineered embryoids mimic post-implantation development in vitro. Nat. Commun..

[9] Langkabel J (2021). Induction of rosette-to-lumen stage embryoids using reprogramming paradigms in ESCs. Nat. Commun..

[10] Amadei G (2021). Inducible stem-cell-derived embryos capture mouse morphogenetic events in vitro. Dev. Cell.

[11] Bao, M. et al. Stem cell-derived synthetic embryos self-assemble by exploiting cadherin codes and cortical tension. *Nat. Cell. Biol.* **24**, 1341–1349 (2022). 10.1038/s41556-022-00984-y PMC9481465 36100738

[12] Sturm K, Tam PP (1993). Isolation and culture of whole postimplantation embryos and germ layer derivatives. Methods Enzymol..

[13] Aguilera-Castrejon A (2021). Ex utero mouse embryogenesis from pre-gastrulation to late organogenesis. Nature.

[14] Zilionis R (2017). Single-cell barcoding and sequencing using droplet microfluidics. Nat. Protoc..

[15] Klein AM (2015). Droplet barcoding for single-cell transcriptomics applied to embryonic stem cells. Cell.

[16] A BJ (2018). The dynamics of gene expression in vertebrate embryogenesis at single-cell resolution. Science.

[17] Pijuan-Sala B (2019). A single-cell molecular map of mouse gastrulation and early organogenesis. Nature.

[18] Simmons DG, Cross JC (2005). Determinants of trophoblast lineage and cell subtype specification in the mouse placenta. Dev. Biol..

[19] Hu D, Cross JC (2010). Development and function of trophoblast giant cells in the rodent placenta. Int. J. Dev. Biol..

[20] Martin, B. K. et al. An optimized protocol for single cell transcriptional profiling by combinatorial indexing. Preprint at *arXiv* https://arxiv.org/abs/2110.15400 (2021).

[21] Qiu C (2022). Systematic reconstruction of cellular trajectories across mouse embryogenesis. Nat. Genet..

[22] Pevny LH, Sockanathan S, Placzek M, Lovell-Badge R (1998). A role for Sox1 in neural determination. Development.

[23] José-Edwards DS (2015). Brachyury, Foxa2 and the *cis*-regulatory origins of the notochord. PLoS Genet..

[24] Kahane N, Kalcheim C (2020). Neural tube development depends on notochord-derived sonic hedgehog released into the sclerotome. Development.

[25] Tadeu AMB, Horsley V (2013). Notch signaling represses p63 expression in the developing surface ectoderm. Development.

[26] Acampora D (2001). OTD/OTX2 functional equivalence depends on 5′ and 3′ UTR-mediated control of Otx2 mRNA for nucleo-cytoplasmic export and epiblast-restricted translation. Development.

[27] Ang S-L, Conlon RA, Jin O, Rossant J (1994). Positive and negative signals from mesoderm regulate the expression of mouse Otx2 in ectoderm explants. Development.

[28] Hettige NC, Ernst C (2019). FOXG1 dose in brain development. Front. Pediatr..

[29] Hu Z-L (2011). The role of the transcription factor Rbpj in the development of dorsal root ganglia. Neural Dev..

[30] Manderfield LJ (2014). Pax3 and Hippo signaling coordinate melanocyte gene expression in neural crest. Cell Rep..

[31] Holz A (2010). The transcription factors Nkx2.2 and Nkx2.9 play a novel role in floor plate development and commissural axon guidance. Development.

[32] Ericson J (1997). Pax6 controls progenitor cell identity and neuronal fate in response to graded Shh signaling. Cell.

[33] Briscoe J (1999). Homeobox gene Nkx2.2 and specification of neuronal identity by graded Sonic hedgehog signalling. Nature.

[34] Novitch BG, Chen AI, Jessell TM (2001). Coordinate regulation of motor neuron subtype identity and pan-neuronal properties by the bHLH repressor Olig2. Neuron.

[35] Ribes V (2010). Distinct Sonic Hedgehog signaling dynamics specify floor plate and ventral neuronal progenitors in the vertebrate neural tube. Genes Dev..

[36] Sasaki H, Hogan BL (1994). HNF-3/l as a regulator of floor plate development. Cell.

[37] Serbedzija GN, Mcmahon AP (1997). Analysis of neural crest cell migration in Splotch mice using a neural crest-specific LacZ reporter. Dev. Biol..

[38] Southard-Smith EM, Kos L, Pavan WJ (1998). Sox10 mutation disrupts neural crest development in Dom Hirschsprung mouse model. Nat. Genet..

[39] la Manno G (2018). RNA velocity of single cells. Nature.

[40] La Manno G (2021). Molecular architecture of the developing mouse brain. Nature.

[41] Gao Z (2010). Ets1 is required for proper migration and differentiation of the cardiac neural crest. Development.

[42] Kaufman MH, Chang HH, Shaw JP (1995). Craniofacial abnormalities in homozygous Small eye (*Sey*/*Sey*) embryos and newborn mice. J. Anat..

[43] Sansom SN (2009). The level of the transcription factor Pax6 is essential for controlling the balance between neural stem cell self-renewal and neurogenesis. PLoS Genet..

[44] Tzouanacou E, Wegener A, Wymeersch FJ, Wilson V, Nicolas JF (2009). Redefining the progression of lineage segregations during mammalian embryogenesis by clonal analysis. Dev. Cell.

[45] Koch F (2017). Antagonistic activities of Sox2 and Brachyury control the fate choice of neuro-mesodermal progenitors. Dev. Cell.

[46] Pourquié, O. in *Current Topics in Developmental Biology* vol. 47 (ed. Ordahl, C. P.) 81–105 (Academic Press, 1999).

[47] Dohn TE (2019). Nr2f-dependent allocation of ventricular cardiomyocyte and pharyngeal muscle progenitors. PLoS Genet..

[48] Bao ZZ, Bruneau BG, Seidman JG, Seidman CE, Cepko CL (1999). Regulation of chamber-specific gene expression in the developing heart by Irx4. Science.

[49] Nowotschin S (2019). The emergent landscape of the mouse gut endoderm at single-cell resolution. Nature.

[50] Lawson KA, Hage WJ (1994). Clonal analysis of the origin of primordial germ cells in the mouse. Ciba Found. Symp..

[51] Saitou M, Barton SC, Surani MA (2002). A molecular programme for the specification of germ cell fate in mice. Nature.

[52] Dobreva MP (2012). Periostin as a biomarker of the amniotic membrane. Stem Cells Int..

[53] Naiche LA, Papaioannou VE (2003). Loss of Tbx4 blocks hindlimb development and affects vascularization and fusion of the allantois. Development.

[54] Yagi S, Shiojiri N (2017). Identification of novel genetic markers for mouse yolk sac cells by using microarray analyses. Placenta.

[55] Tanaka Y (2014). Circulation-independent differentiation pathway from extraembryonic mesoderm toward hematopoietic stem cells via hemogenic angioblasts. Cell Rep..

[56] Latos PA, Hemberger M (2016). From the stem of the placental tree: trophoblast stem cells and their progeny. Development.

[57] Tarazi S (2022). Post-gastrulation synthetic embryos generated ex utero from mouse naïve ESCs. Cell.

[58] Lau, K. Y. C. et al. Mouse embryo model derived exclusively from embryonic stem cells undergo neurulation and heart development. *Cell Stem Cell*10.1016/j.stem.2022.08.013 (2022). 10.1016/j.stem.2022.08.013 PMC9648694 36084657

[59] Rhee JM (2006). In vivo imaging and differential localization of lipid-modified GFP-variant fusions in embryonic stem cells and mice. Genesis.

[60] Egli D, Rosains J, Birkhoff G, Eggan K (2007). Developmental reprogramming after chromosome transfer into mitotic mouse zygotes. Nature.

[61] Mesnard D, Filipe M, Belo JA, Zernicka-Goetz M (2004). The anterior–posterior axis emerges respecting the morphology of the mouse embryo that changes and aligns with the uterus before gastrulation. Curr. Biol..

[62] Alsanie WF (2017). Specification of murine ground state pluripotent stem cells to regional neuronal populations. Sci. Rep..

[63] Amadei, G. et al. Stem cell-derived mouse embryos develop within an extra-embryonic yolk sac to form anterior brain regions and a beating heart-like structure. *Protocol Exchange*10.21203/rs.3.pex-2006/v1 (2022).

[64] Bedzhov I, Leung CY, Bialecka M, Zernicka-Goetz M (2014). In vitro culture of mouse blastocysts beyond the implantation stages. Nat. Protoc..

[65] Parekh S, Ziegenhain C, Vieth B, Enard W, Hellmann I (2018). zUMIs—a fast and flexible pipeline to process RNA sequencing data with UMIs. Gigascience.

[66] Stuart T (2019). Comprehensive integration of single-cell data. Cell.

[67] Wolock SL, Lopez R, Klein AM (2019). Scrublet: computational identification of cell doublets in single-cell transcriptomic data. Cell Syst..

[68] Korsunsky I (2019). Fast, sensitive and accurate integration of single-cell data with Harmony. Nat. Methods.

[69] Wolf FA, Angerer P, Theis FJ (2018). SCANPY: large-scale single-cell gene expression data analysis. Genome Biol..

[70] Bergen V, Lange M, Peidli S, Wolf FA, Theis FJ (2020). Generalizing RNA velocity to transient cell states through dynamical modeling. Nat. Biotechnol..

[71] Cao J (2019). The single-cell transcriptional landscape of mammalian organogenesis. Nature.

[72] Renaud G, Stenzel U, Maricic T, Wiebe V, Kelso J (2015). deML: robust demultiplexing of Illumina sequences using a likelihood-based approach. Bioinformatics.

[73] Cao J (2017). Comprehensive single-cell transcriptional profiling of a multicellular organism. Science.

[74] Dobin A (2013). STAR: ultrafast universal RNA-seq aligner. Bioinformatics.

[75] Anders S, Pyl PT, Huber W (2015). HTSeq–a Python framework to work with high-throughput sequencing data. Bioinformatics.

[76] Schindelin J (2012). Fiji: an open-source platform for biological-image analysis. Nat. Methods.

[77] Boulanger J (2010). Patch-based nonlocal functional for denoising fluorescence microscopy image sequences. IEEE Trans. Med. Imaging.

[78] Takei Y (2021). Integrated spatial genomics reveals global architecture of single nuclei. Nature.

[79] Huang DW, Sherman BT, Lempicki RA (2009). Systematic and integrative analysis of large gene lists using DAVID bioinformatics resources. Nat. Protoc..

[80] Sherman BT (2022). DAVID: a web server for functional enrichment analysis and functional annotation of gene lists (2021 update). Nucleic Acids Res..

[81] Consortium TGO (2021). The Gene Ontology resource: enriching a GOld mine. Nucleic Acids Res..

[82] Mi H, Muruganujan A, Ebert D, Huang X, Thomas PD (2019). PANTHER version 14: more genomes, a new PANTHER GO-slim and improvements in enrichment analysis tools. Nucleic Acids Res..

[83] Ashburner M (2000). Gene Ontology: tool for the unification of biology. Nat. Genet..

