# Supplementary material for: Embryo model completes gastrulation to neurulation and organogenesis
Source: Nature. 2022 Aug 25;610(7930):143–53. doi: 10.1038/s41586-022-05246-3 (PMC9534772; doi:10.1038/s41586-022-05246-3)
Supplement: Supplementary file 1 — Reporting Summary [file 41586_2022_5246_MOESM1_ESM.pdf]

nature portfolio

Corresponding author(s): Magdalena Zernicka-Goetz

Last updated by author(s): Aug 12, 2022

Reporting Summary

Nature Portfolio wishes to improve the reproducibility of the work that we publish. This form provides structure for consistency and transparency in reporting. For further information on Nature Portfolio policies, see our Editorial Policies and the Editorial Policy Checklist.

Statistics

For all statistical analyses, confirm that the following items are present in the figure legend, table legend, main text, or Methods section.

n/a	Confirmed	
☐	☒	The exact sample size (*n*) for each experimental group/condition, given as a discrete number and unit of measurement
☐	☒	A statement on whether measurements were taken from distinct samples or whether the same sample was measured repeatedly
☐	☒	The statistical test(s) used AND whether they are one- or two-sided<br>*Only common tests should be described solely by name; describe more complex techniques in the Methods section.*
☒	☐	A description of all covariates tested
☐	☒	A description of any assumptions or corrections, such as tests of normality and adjustment for multiple comparisons
☐	☒	A full description of the statistical parameters including central tendency (e.g. means) or other basic estimates (e.g. regression coefficient) AND variation (e.g. standard deviation) or associated estimates of uncertainty (e.g. confidence intervals)
☐	☒	For null hypothesis testing, the test statistic (e.g. *F*, *t*, *r*) with confidence intervals, effect sizes, degrees of freedom and *P* value noted<br>*Give P values as exact values whenever suitable.*
☒	☐	For Bayesian analysis, information on the choice of priors and Markov chain Monte Carlo settings
☒	☐	For hierarchical and complex designs, identification of the appropriate level for tests and full reporting of outcomes
☐	☒	Estimates of effect sizes (e.g. Cohen's *d*, Pearson's *r*), indicating how they were calculated

Our web collection on statistics for biologists contains articles on many of the points above.

Software and code

Policy information about availability of computer code

Data collection	For the inDrops sequencing, the following tools were used: the BCL files were converted to Fastq files using Illumina's bcl2fastq software 2.20. The sequenced libraries were quality-inspected using the FastQC v0.11.9 tool and de-multiplexed using the Pheniqs tool from biosails v2.1.0. The fastq files were further filtered, mapped to a mouse GRCm38.99 reference genome with GRCm38.99 gtf annotation and deduplicated using the zUMIs 2.9.7 pipeline. The count matrices with exonic and intronic counts were then used as an input for downstream analysis using Seurat version 3. Doublet scores computed using Scrublet v 0.1. The natural and synthetic embryo datasets were integrated in Seurat v3 and shared embeddings were corrected for batch effect (both systems and timepoints collected) using Harmony v4.3.12. Louvain clustering was performed on the shared embeddings and markers, computed using the FindAllMarkers function from Seurat, were used to annotate cell types. Pearson correlation coefficients between cell types for each system, single-cell velocity profiles and latent times were computed using the Scanpy v1.6.0 and scVelo tools v.0.2.4. These tools are open source<br>For the tiny-sci sequencing analysis, the following common, freely available data analysis software were used in this project: bcl2fastq version 2.20 (https://support.illumina.com), deML version 1.1.3 (https://github.com/grenaud/deML), HTseq version 0.6.1 (https://github.com/htseq/htseq), trim_galore version 0.6.5 (https://github.com/FelixKrueger/TrimGalore), STAR version 2.6.1d (https://github.com/alexdobin/STAR), scrublet version 0.1 (https://github.com/swolock/scrublet), Scanpy version 1.6.0 (https://github.com/theislab/scanpy), Monocle version 2, 3, and 3-alpha (https://cole-trapnell-lab.github.io/monocle3), Seurat version 3 (https://github.com/satijalab/seurat), ggplot2 version 3.3.5 (https://ggplot2.tidyverse.org/).
Data analysis	Open Source: Fiji 1.52, NDSAFIR 3.0, Smart Denoise Plugin (Gurdon Institute)<br>Commercial Software: Adobe Illustrator 26.0.1, Prism GraphPad v9.2 and Microsoft Excel

For manuscripts utilizing custom algorithms or software that are central to the research but not yet described in published literature, software must be made available to editors and reviewers. We strongly encourage code deposition in a community repository (e.g. GitHub). See the Nature Portfolio guidelines for submitting code & software for further information.

Data

Policy information about availability of data

All manuscripts must include a data availability statement. This statement should provide the following information, where applicable:

- Accession codes, unique identifiers, or web links for publicly available datasets
- A description of any restrictions on data availability
- For clinical datasets or third party data, please ensure that the statement adheres to our policy

Data Availability

All unique/stable reagents generated in this study are available from the Lead Contact with a completed Materials Transfer Agreement. Source data are provided with this paper. Source data pertaining to graphs that are not compatible with excel formatting can be generated with the raw sequencing data and code provided. Raw single cell sequencing data generated by this work have been deposited in the NCBI Gene Expression Omnibus database (https://www.ncbi.nlm.nih.gov/geo/) and are accessible through the following accession numbers:
The inDrops sc-RNA-sequencing dataset is available at "GSE189425".
The tiny sci-RNA-sequencing dataset is available at "GSE209792" .
The code used to analyse the inDrops sc-RNA-sequencing data is available here: https://github.com/fhlab/scRNAseq_inducedETX
The code used to analyse the tiny sci-RNA-sequencing data is available here: https://github.com/ChengxiangQiu/ETiX_Amadei

Human research participants

Policy information about studies involving human research participants and Sex and Gender in Research.

Reporting on sex and gender	Not applicable
Population characteristics	Not applicable
Recruitment	Not applicable
Ethics oversight	Not applicable

Note that full information on the approval of the study protocol must also be provided in the manuscript.

Field-specific reporting

Please select the one below that is the best fit for your research. If you are not sure, read the appropriate sections before making your selection.

☒ Life sciences ☐ Behavioural & social sciences ☐ Ecological, evolutionary & environmental sciences

For a reference copy of the document with all sections, see nature.com/documents/nr-reporting-summary-flat.pdf

Life sciences study design

All studies must disclose on these points even when the disclosure is negative.

Sample size	The sample size was not predetermined. Sample size was selected on the basis of similar published studies performed by our group and others (Harrison et al., 2017; Sozen et al. 2019; Amadei et al., 2021)
Data exclusions	Synthetic embryos that did not conform to this categories (see below) were excluded from culture and therefore were not analyzed (see below). All ETiX-embryoids were collected from AggreWell for analysis at 4 days of development and analysed under a stereo microscope. In all instances, we selected ETiX-embryoids with cylindrical morphology and two clearly defined cellular compartments surrounded by an outer cell layer. We expect the ESC compartment to be epithelialized with a lumen. The TSC compartment is more variable in appearance and therefore, even though one would also want an epithelial-looking TSC compartment similar to the extra-embryonic ectoderm of natural embryos, we select a wider range of appearances for the TSC compartment. Since the majority of ETiX-embryoids in this study were generated by using wild-type stem cell lines, the selection was based on morphology alone. ETiX-embryoids with the correct body plan of ESC and TSC compartments surrounded by a visceral endoderm-like layer are then transferred to equilibrated media to continue their culture. When selecting at D5, however, we included additional criteria: i) we expect the lumen of the ESC and TSC compartment to be merged; ii) ideally we can observe the beginning of gastrulation on one side of the ETiX-embryoid; iii) we expected the AVE to have migrated to the ESC-TSC boundary and be opposite to the forming streak; iv) ETiX with the AVE stuck at the tip of the structure or not at the boundary were excluded.
Replication	Experiments were repeated as appropriate with different cell lines to ensure that the results are cell line independent.<br>For the experiments reported herein we were able to successfully generate ETiX-embryoids with the following ESC lines:<br>• Wildtype CD1 ESCs<br>• Sox2-Venus/Brachyury-mCherry/Oct4-ECFP ESCs

• CAG-GFP/tetO-mCherry ESCs
• Blimp1-GFP ESCs
• BVSC ESCs
In addition to the lines indicated above, we also tried five more that could progress to day 5 and 6 but not beyond.
These lines were:
• Lfng reporter (LuVeLu) ESCs (a gift from Dr. Alexander Aulelha and Dr. Ina Sonnen)
• Msgn1-Venus ESCs (a gift from Prof. Olivier Pourquié)
• Hes7-Achilles ESCs (a gift from Prof. Olivier Pourquié)
• Sox1-GFP ESCs (a gift from Prof. Austin Smith)
• mTmG ESCs (generated by us in-house)
No results reported in this study were generated with these 5 unsuccessful lines.
The majority of the structures presented in this study were generated using wildtype CD1 ESCs, wildtype CD1 TSCs and CD1/tetO-Gata4 ESCs.
All experiments in this study were repeated for a minimum of 2 times, although the majority was repeated for three times or more. The only exception to this is the sequential smFISH which was performed once an a natural embryo and once on an ETiX-embryoid due to the time required for one run. We have found that batches of serum of suboptimal quality and suboptimal cell culture conditions can lead to unsuccessful experiments, hence not all attempts at replication were successful. 7% of all experiments attempted were not successful.

Randomization

Synthetic embryos and natural embryos were randomly allocated for analysis in the panels presented in the study

Blinding

The authors were not blind during the study because they knew which samples were natural embryos (ie they were recovered and cultured from the mother) vs ETiX-embryoids that were grown from stem cells.

Reporting for specific materials, systems and methods

We require information from authors about some types of materials, experimental systems and methods used in many studies. Here, indicate whether each material, system or method listed is relevant to your study. If you are not sure if a list item applies to your research, read the appropriate section before selecting a response.

Materials & experimental systems

n/a	Involved in the study
☐	☒ Antibodies
☐	☒ Eukaryotic cell lines
☒	☐ Palaeontology and archaeology
☐	☒ Animals and other organisms
☒	☐ Clinical data
☒	☐ Dual use research of concern

Methods

n/a	Involved in the study
☒	☐ ChIP-seq
☒	☐ Flow cytometry
☒	☐ MRI-based neuroimaging

Antibodies

Antibodies used

Rabbit polyclonal anti-GATA-4 Santa Cruz Biotechnology Cat# sc-9053, RRID:AB_2247396 Dilution: 1:500

Goat polyclonal anti-GATA-6 R&D Systems Cat# AF1700, RRID:AB_2108901 Dilution: 1:500
Goat polyclonal anti-Otx2 R&D Systems Cat# AF1979, RRID:AB_2157172 Dilution:1:500
Rabbit monoclonal anti-RUNX1 / AML1 Abcam Cat# ab92336, RRID:AB_2049267 Dilution 1:500 Clone EPR3099
Goat polyclonal anti-Sox17 R&D Systems Cat# AF1924, RRID:AB_355060 Dilution: 1:200
Rabbit monoclonal anti-HoxB4 Abcam Cat# ab133521 Dilution: 1:500 Clone EPR1917
Rabbit polyclonal anti-Sox1 Cell Signaling Technology Cat# 4194 Dilution: 1:500 RRID:AB_1904140

Goat polyclonal anti-Sox1 R&D Systems Cat# AF3369 RRID:AB_2239879 Dilution: 1:200

Goat polyclonal anti-Brachyury R&D Systems Cat# AF2085, RRID:AB_2200235 Dilution: 1:500
Mouse monoclonal anti-Sox2 Santa Cruz Biotechnology Cat# sc-365823 RRID:AB_10842165 Clone E4 Dilution: 1:500

Rat monoclonal anti-Sox2 Thermo Fisher Scientific Cat# 14-9811-82 RRID:AB_11219471 Clone Btjce Dilution 1:500

Rabbit monoclonal anti-FoxG1 Abcam Cat# ab196868 RRID:AB_2892604 clone EPR18987 Dilution 1:500

Rabbit monoclonal anti-FoxA2 / HNF3 Cell Signaling Technology Cat# 8186, RRID:AB_10891055 Clone D56D6 Dilution 1:200
Mouse monoclonal anti-Myh2 R&D Systems Cat# MAB4470 RRID:AB_1293549 Clone MF20 Dilution: 1:500

Goat polyclonal anti-Nkx2.5 R&D Systems Cat# AF2444 RRID:AB_355269 Dilution 1:500

Rabbit polyclonal anti-Nanog Abcam Cat# ab80892 RRID:AB_2150114 Dilution 1:500

Mouse monoclonal anti-Pax3 DSHB Cat# AB_528426 RRID:AB_528426 Dilution 1:500 Clone MIgG2a

Rabbit polyclonal anti-Pax6 BioLegend Cat# PRB-278P RRID:AB_291612 Dilution 1:500

Rabbit polyclonal anti-Olig2 IBL America Cat# 18953 RRID:AB_1630817 Dilution 1:500

Mouse monoclonal anti Nkx2.2 DSHB Cat# 74.5A5 RRID:AB_531794 Clone: MIgG2b Dilution 1:500

Mouse monoclonal anti-Sox10 Santa Cruz Biotechnology Cat# sc-365692 RRID:AB_10844002 Clone A-2, Dilution: 1:500

Goat polyclonal anti-Stella R&D Systems Cat# AF2566 RRID:AB_2094147 Dilution: 1:500

Alexa Fluor® 488 Phalloidin Thermo Fisher Scientific Cat# A12379 Dilution 1:1000

Alexa Fluor® 594 Phalloidin Thermo Fisher Scientific Cat# A12381 Dilution 1:1000

Alexa Fluor® 647 Phalloidin Thermo Fisher Scientific Cat# A22287 Dilution 1:1000

Donkey anti-Mouse IgG (H+L) Highly Cross-Adsorbed Secondary Antibody, Alexa Fluor 488 Thermo Fisher Scientific Cat# A-21202, RRID:AB_141607 Dilution: 1:500
Donkey anti-Rabbit IgG (H+L) Highly Cross-Adsorbed Secondary Antibody, Alexa Fluor 488 Thermo Fisher Scientific Cat# A-21206, RRID:AB_2535792 Dilution: 1:500
Donkey anti-Goat IgG (H+L) Cross-Adsorbed Secondary Antibody, Alexa Fluor 488 Thermo Fisher Scientific Cat# A-11055, RRID:AB_2534102 Dilution: 1:500
Donkey anti-Mouse IgG (H+L) Highly Cross-Adsorbed Secondary Antibody, Alexa Fluor 568 Thermo Fisher Scientific Cat# A10037, RRID:AB_2534013 Dilution: 1:500
Donkey anti-Rabbit IgG (H+L) Highly Cross-Adsorbed Secondary Antibody, Alexa Fluor 568 Thermo Fisher Scientific Cat# A10042, RRID:AB_2534017 Dilution: 1:500
Donkey anti-Goat IgG (H+L) Cross-Adsorbed Secondary Antibody, Alexa Fluor 568 Thermo Fisher Scientific Cat# A-11057, RRID:AB_2534104 Dilution: 1:500
Donkey anti-Mouse IgG (H+L) Highly Cross-Adsorbed Secondary Antibody, Alexa Fluor 647 Thermo Fisher Scientific Cat# A-31571, RRID:AB_162542 Dilution: 1:500
Donkey anti-Rabbit IgG (H+L) Highly Cross-Adsorbed Secondary Antibody, Alexa Fluor 647 Thermo Fisher Scientific Cat# A-31573, RRID:AB_2536183 Dilution: 1:500
Donkey anti-Goat IgG (H+L) Cross-Adsorbed Secondary Antibody, Alexa Fluor 647 Thermo Fisher Scientific Cat# A-21447, RRID:AB_2535864 Dilution: 1:500
Donkey Anti-Rat IgG H&L (Alexa Fluor® 647) preadsorbed antibody Abcam Cat# ab150155, RRID:AB_2813835 DIlution: 1:500

Validation

All the antibodies have been validated by the company that sells them. Details of the validation statement, antibody profiles and relevant citations can be found on the manufacturer's website. In addition to that we validated all antibodies used in this study by immunofluorescence in natural mouse embryos and cross-checked them against their well-described expression pattern in reported studies.

Eukaryotic cell lines

Policy information about cell lines and Sex and Gender in Research

Cell line source(s)

Cell Lines and Culture Conditions
Cell lines used in this study were mouse cell lines and include:
• CAG-GFP/tetO-mCherry mouse ESCs (constitutive GFP expression in the membrane; transient mCherry expression upon Dox treatment). The parent CAG-GFP/tetO-mCherry ESC line was derived from an existing mouse line with constitutive CAG-GFP expression and Dox-induced transient mCherry expression. This line was generated by breeding CAG-GFP reporter mice59 and tetO-mCherry Histone mice60. For the purpose of this study, an independent Dox-inducible Gata4-expressing cassette was introduced into the CAG-GFP/tetO-mCherry ESC line by piggyBac-based transposition, as described below, thus mCherry and Gata4 are regulated by two, independent Dox-responsive promoters.

• CAG-GFP/tetO-mCherry/tetO-Gata4 ESCs generated in-house.
• Cerl-GFP ESCs (GFP expression under the control of the Cerl-promoter) were derived from a published Cerl-GFP mouse line (Mesnard et al. 2004).
• Cerl-GFP/tetO-Gata4 ESCs generated in-house.
• Wildtype CD1 TSCs generated in-house.
• Wildtype CD1 ESCs (a gift from Dr. Jenny Nichols)
• CD1/tetO-Gata4 ESCs were generated in-house
• Sox2-Venus/Brachyury-mCherry/Oct4-ECFP ESCs (a gift from Dr. Jesse Veenvliet and Prof. Bernhard G. Hermann)
• Blimp1-GFP ESCs (a gift from Prof. Azim Surani)
• BVSC ESCs (a gift from Prof. Wolf Reik)
For the experiments reported herein we were able to successfully generate ETiX-embryoids with the following ESC lines:
• Wildtype CD1 ESCs
• Sox2-Venus/Brachyury-mCherry/Oct4-ECFP ESCs
• CAG-GFP/tetO-mCherry ESCs
• Blimp1-GFP ESCs
• BVSC ESCs
In addition to the lines indicated above, we also tried five more that could progress to day 5 and 6 but not beyond.
These lines were:
• Lfng reporter (LuVeLu) ESCs (a gift from Dr. Alexander Aulelha and Dr. Ina Sonnen)
• Msgn1-Venus ESCs (a gift from Prof. Olivier Pourquié)

• Hes7-Achilles ESCs (a gift from Prof. Olivier Pourquié)
• Sox1-GFP ESCs (a gift from Prof. Austin Smith)
• mTmG ESCs (generated by us in-house)
No results reported in this study were generated with these 5 unsuccessful lines.
The majority of the structures presented in this study were generated using wildtype CD1 ESCs, wildtype CD1 TSCs and CD1/tetO-Gata4 ESCs.

Authentication	The cells lines were not authenticated
Mycoplasma contamination	Cell lines were routinely screened every two weeks for mycoplasma contamination and all tested negative throughout this study
Commonly misidentified lines (See ICLAC register)	None were used in this study

Animals and other research organisms

Policy information about studies involving animals; ARRIVE guidelines recommended for reporting animal research, and Sex and Gender in Research

Laboratory animals	Mice (six-week-old CD-1 males from Charles River and transgenic females bred in house) used in the experiments were kept in animal house, following national and international guidelines. All experiments performed were under the regulation of the Animals (Scientific Procedures) Act 1986 Amendment Regulations 2012 and were reviewed by the University of Cambridge Animal Welfare and Ethical Review Body (AWERB). Experiments were also approved by the Home Office. Animals
Wild animals	No wild animals were used in this study
Reporting on sex	Both male and female embryos were used in this study; the embryos were not genotyped to determine the sex
Field-collected samples	No field collected samples were used in this study
Ethics oversight	All experiments performed were under the regulation of the Animals (Scientific Procedures) Act 1986 Amendment Regulations 2012 and were reviewed by the University of Cambridge Animal Welfare and Ethical Review Body (AWERB). Experiments were also approved by the Home Office.

Note that full information on the approval of the study protocol must also be provided in the manuscript.
